# High fiber diet mitigates exposure risk of nanoplastics in male mice

**DOI:** 10.1038/s41467-026-78675-7

**Published:** 2026-10-15

**Authors:** Zimin Yu, Gulizhabaier Abulipizi, Yanlun Fang, Chaomin Qu, Leping Ding, Ziang Zong, Jie Huang, Ziyang Chen, Juanjuan Zhou, Zhanjun Li, Eddy Y. Zeng

**Affiliations:** 1 School of Biomedical Engineering, Guangzhou Medical University, Guangzhou, China; 2 School of Public Health, Guangdong Medical University, Dongguan, China; 3 Institute of Environmental Health, School of Environment and Energy, South China University of Technology, Guangzhou, China

**Keywords:** Environmental impact, Gastroenterology, Small molecules

## Abstract

Potential health risk associated with dietary intake of nanoplastics has emerged as a global health concern, yet approaches for reducing gastrointestinal nanoplastic exposure remain poorly explored. Here, we investigated whether increasing dietary fiber intake influences the gastrointestinal fate of nanoplastics in male mice utilizing a visible/near-infrared dual fluorescence labeling technique combined with non-invasive imaging. The estimated retention rates of nanoplastics-associated fluorescence are decreased by two and seven times in male mice fed with medium fiber diet and high fiber diet compared to those with standard fiber diet, 24 h after oral intake. High fiber diet is associated with attenuated nanoplastics-induced epithelial damage to intestinal tract and declined detectable NIR fluorescence in important organs such as liver during 28-d oral exposure to nanoplastics. In vitro simulations show that dietary fiber physically encapsulates and adheres nanoplastics to decrease their bioavailability. Understanding how dietary constituents influence the gastrointestinal fate of nanoplastics may provide a scientific basis for the future development of dietary strategies aimed at reducing nanoplastic exposure.

## Abstract (web summary)

Dietary exposure to nanoplastics is an emerging health concern. Here, the authors show that high-fiber diet reduces nanoplastic gastrointestinal retention, alleviates intestinal injury, and limits organ translocation in male mice.

## Introduction

Plastic pollution poses serious threats to both ecosystem and human health^1^. Annual discharge of plastics to the environment is estimated at up to millions of metric tons, and is experiencing an increase of 1.5 to 2.5 times by 2040^2^. Even if anthropogenic plastic discharge is ceased immediately, continuing fragmentation or breakdown of existing plastic debris would propel the proliferation of microplastics (MPs; plastic debris smaller than 5 mm) and nanoplastics (NPs; <1 μm) for hundreds of years, forging persistent threats to human health. Microplastics with larger particle sizes can be more readily removed from consumer products. However, MPs can fragment to much more invisible NPs and have been frequently found in ecosystems and human diets^3,4^.

Dietary intake is the main pathway of MPs and NPs invading into our body^5,6^. For example, NPs have been frequently found in human feces and pathological samples, suggesting that NPs could accumulate in gastrointestinal (GI) tract, lead to mechanical damage and inflammation to GI mucosa, and sorb to the circulation system^7,8^. Accumulated NPs could also discharge diverse toxic pollutants, resulting in imbalanced intestinal microbiota and adversely impacting immune system functionality^9,10^. If NPs are not rapidly cleared from the GI tract, prolonged retention may allow them to translocate across the intestinal barrier. Potential effects could escalate from chronic inflammation to severe health consequences^11^, such as cardiovascular diseases^7^, stroke^12^, cerebral thrombosis^13^, and Alzheimer’s disease^14^.

Dietary fiber, a complex carbohydrate polymer consisting of more than three monosaccharide units, resists digestion by endogenous enzymes. High fiber diet, coupled with nutritional interventions, has been proved effective in both prevention and treatment of diseases that are correlated with environmental pollution^15,16^. Growing evidence has supported the notion that dietary fiber could decrease systemic inflammation^17^, foster the proliferation of beneficial bacteria^18^, modulate immune responses^19^, and preserve the integrity of intestinal barrier^20^. Dietary fiber is also able to augment stool bulk and enhance intestinal motility, shortening the contact time between intestinal mucosal cells and environmental pollutants, such as perfluorooctane sulfonate^15^, perfluoroalkyl acids^21^, and heavy metals^22^. We therefore hypothesize that a high fiber diet would be able to diminish accumulation of NPs in GI tract.

Herein we verify the hypothesis that a high-fiber diet modulates the fate of near-infrared (NIR) NPs (aged polystyrene (PS) NPs labeled with 1,1’-dioctadecyl-3,3,3’,3’-tetramethylindodicarbocyanine,4-chlorobenzenesulfonate salt (DiD; red fluorescence) and 1,1-dioctadecyl-3,3,3,3-tetramethylindotricarbocyaine iodide (DiR; NIR fluorescence) by assessing the excretion and bioaccumulation of NPs and preliminary health effects in a living mouse model. Upon short-term (7 days) and long-term (28 days) dietary intervention (see Methods), the spatiotemporal biodistribution of NIR-NPs was systematically tracked through non-invasive NIR fluorescence imaging at multiple time points. Excretion and accumulation of NPs were examined with fluorescence assays, complemented by histological, immunofluorescence, and ultrastructural analyses to assess intestinal tissue alterations and NPs distribution. In addition, in vitro simulated digestion was performed to explore the interaction between dietary fiber and NPs. The present study highlights the potential influence of dietary composition on the gastrointestinal fate of NPs and provides a basis for future studies of diet-based approaches to reducing NPs exposure.

## Results

### Fluorescence labeling of nanoplastics

**Fig. 1:**
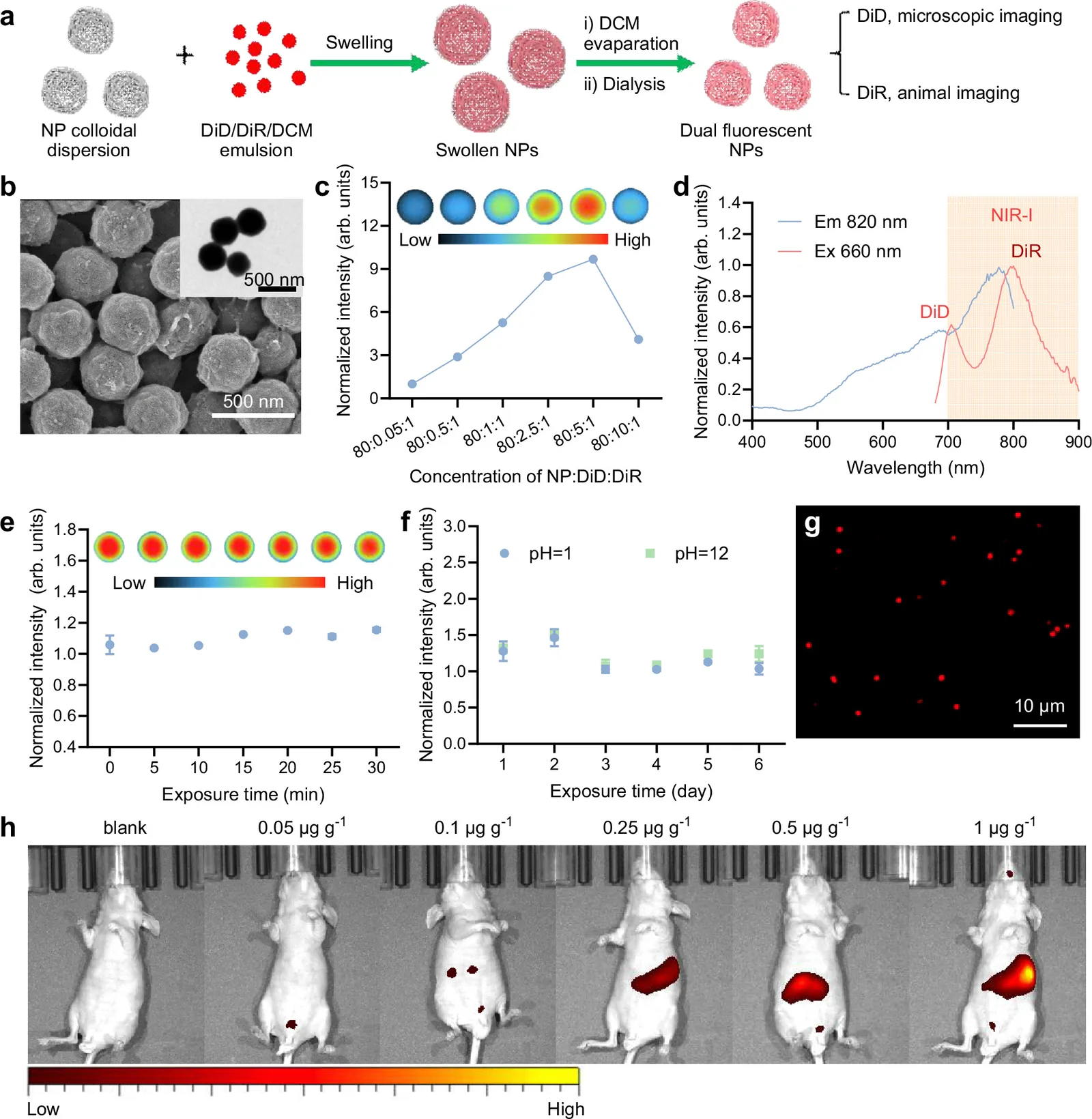
Characteristic of aged near-infrared nanoplastics (NIR-NPs). **a** Scheme of the synthesis process of dual fluorescent NPs; **b** scanning electron microscopy and transmission electron microscopy images of NIR-NPs; **c** optimal ratio of aged NPs to DiD and DiR; **d** photoluminescence spectrum of NIR-NPs; **e** fluorescence stability during continuous 30 min 680 nm laser excitation; **f** fluorescence stability during continuous exposure in solution with pH 1 and 12 for 6 days; **g** single particle fluorescence imaging of NIR-NPs; and **h** NIR images of different oral doses of NIR-NPs in male mice; representative images are shown from three biologically independent male mice per group with similar results. Data were presented as average ± standard error (*n* = 3 independent samples). Source data are provided as a Source Data file.

An emulsion-solvent swelling method was used to encapsulate fluorescent dyes inside the NIR-NPs (Fig. 1a). Free dyes and residue impurities were removed by dialysis. Notably, only encapsulated dyes have fluorescence which is severely quenched by water. The as-synthesized NIR-NPs have a spherical morphology (Fig. 1b), a diameter of 284 ± 15 nm and a zeta potential of – 9.2 ± 0.6 mV. The fluorescence property was optimized by adjusting the mass ratio of NPs:DiD:DiR (Fig. 1c). The optimized NIR-NPs have a visible emission at ~700 nm and a NIR emission at ~800 nm under 680 nm excitation, both of which shift towards longer wavelengths than those of DiD (663 nm) and DiR (780 nm), respectively (Fig. 1d).

As dye molecules were encapsulated inside NP particles and thus protected from outside interfering factors (e.g., oxygen and water), no fluorescence decay or significant photobleaching was observed after a continuous red-light excitation for 30 min (Fig. 1e). NIR-NPs also showed stability at extreme pH values (pH 1 and 12) for 6 d (Fig. 1f); hence they can endure harsh conditions induced by gastric acid and other digestive enzymes in GI. Benefitting from intense fluorescence, single particle fluorescence imaging of NIR-NPs was realized under confocal microscopic observation (Fig. 1g). In vivo imaging in male mice showed low autofluorescence in the NIR-I window and distinct NP fluorescence signals as low as 0.1 μg g^–1^ (NIR-NPs per mouse body weight) (Fig. 1h). All these attributes indicate that the as-synthesized NIR-NPs are able to resist physical and chemical quenching under harsh conditions, and suitable for non-invasive tracking of NP excretion after dietary intervention in long-term animal GI exposure.

### Dietary fiber facilitates excretion of nanoplastics

**Fig. 2:**
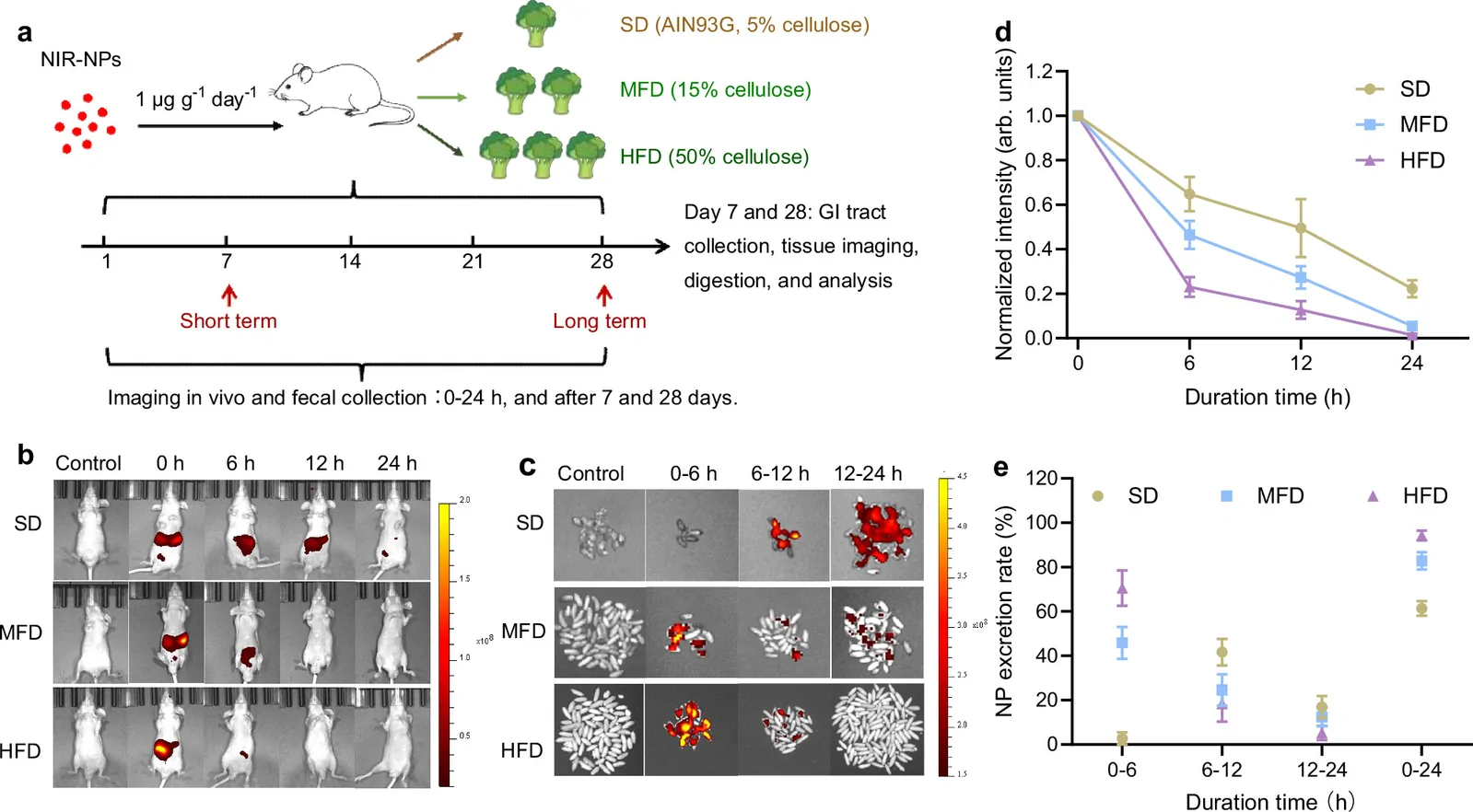
A high-fiber diet promotes rapid excretion of near-infrared nanoplastics (NIR-NPs). **a** Overview of the experimental design; **b** dynamic biodistribution of NIR-NPs following standard diet (SD), medium fiber diet (MFD), and high fiber diet (HFD) interventions during two day period; **c** NIR-NPs in feces following SD, MFD, and HFD interventions; **d** normalized intensity of NIR-NPs following SD, MFD, and HFD interventions; and **e** excretion rates of NIR-NPs following SD, MFD, and HFD intervention; representative images are shown from six biologically independent male mice per group with similar results. Data are expressed as average ± standard error (*n* = 6 biological replicates). Source data are provided as a Source Data file.

Various NP fluorescence decay patterns were observed across dietary regimens (Fig. 2a, b). NIR-NPs were also detected in fecal matter (Fig. 2c), aligning with in vivo imaging results. Male mice receiving standard fiber diet (5%; SD) feed exhibited a slow NIR-NPs excretion pattern, characterized by gradually decreasing fluorescence intensity from 1.0 to 0.65 ± 0.07 during the 0–6 h post gavage period, which amounts to a retention rate of 65% for NIR-NPs 6 h after oral intake, and residual NIR-NPs remained detectable even after 24 h (Fig. 2d and Table S1). Both medium fiber (15%; MFD) and high fiber (50%; HFD) diet feed groups rapidly excreted NIR-NPs. Within the first 0–6 h post-gavage, fluorescence intensity decreased sharply from 1.0 to 0.46 ± 0.06 in the MFD group and from 1.0 to 0.23 ± 0.04 in the HFD group (Fig. 2d and Table S1). These results indicate that the NIR-NPs retention rates in male mice GI decreased from 65% in the SD feed group to 46% and 23% in the MFD and HFD groups, respectively, 6 h after oral intake. At 12 h after NIR-NPs intake, the NIR-NPs signals were markedly decreased in both MFD and HFD-fed male mice (Fig. 2b). Quantitatively, MFD and HFD significantly accelerated NIR-NPs excretion within the first 6 h (46 ± 6.9% and 71 ± 7.6%, respectively) compared to the SD group (2.8 ± 2.6%) (Fig. 2e and Table S2). The total amount of NIR-NPs recovered from feces within 0–24 h was higher in the MFD (17 ± 0.76 μg) and HFD (19 ± 0.42 μg) groups than in the SD group (12 ± 0.64 μg) (Fig. S1). After 24 h of NIR-NPs intake, the total NIR-NPs excretion rates were 61 ± 3.2% (SD), 83 ± 3.8% (MFD), and 94 ± 2.1% (HFD) (Fig. 2e and Table S2). In other words, the accumulated NP rate in male mice fed with SD (39%) was about 2 and 7 times those in male mice fed with MFD (17%) and HFD (6%), respectively.

### Dietary intervention modulates accumulation of nanoplastics

**Fig. 3:**
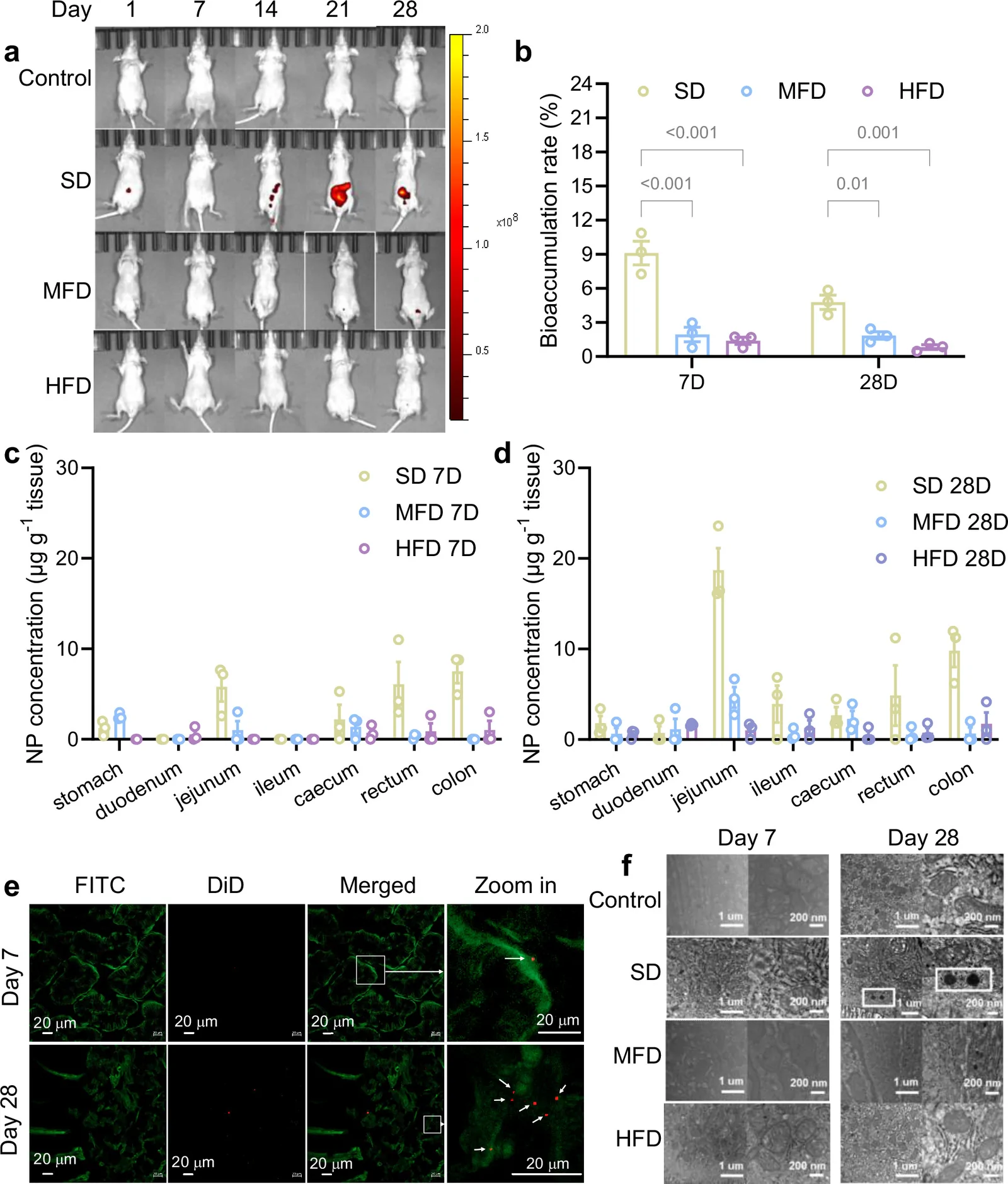
Biodistribution of nanoplastics (NPs) following standard diet (SD), medium fiber diet (MFD), and high fiber diet (HFD) interventions. **a** Dynamic biodistribution of NPs following SD, MFD, and HFD interventions after 28-d digestion; **b** bioaccumulation rate of NPs in the gastrointestinal tract after 7 and 28 consecutive days of diet intervention; statistical analysis was performed through two-way ANOVA followed by Tukey’s multiple comparison tests, and significant differences (*p* < 0.05) are indicated above the bars; **c** accumulation concentration of NPs in the gastrointestinal tract after 7 consecutive days and **d** 28 days of diet intervention; data are expressed as average ± standard error from three biological replicates. **e** laser confocal images show NPs fluorescent signal in SD group after 7 and 28-day exposure; and **f** transmission electron microscope images show NPs located in ileal tissues in SD group after 28-day exposure; representative images are shown from three biologically independent male mice per group with similar results. Source data are provided as a Source Data file.

The time-dependent translocation and accumulation of NIR-NPs in all feeding groups were visualized through serial in vivo imaging (Fig. 3a). The accumulation rate of NIR-NPs was significantly higher in the SD group (9.1 ± 0.85%) than in the MFD (2.0 ± 0.52%) and HFD groups (1.4 ± 0.26%) upon short-term (7 d) dietary intervention (*p* < 0.05; Fig. 3b and Table S3). After long-term (28 days) intervention, intestinal accumulation of NPs in the SD group decreased to 4.8 ± 0.51% compared to short-term exposure but remained significantly higher than those in the MFD (1.9 ± 0.27%) and HFD (0.84 ± 0.17%) groups (*p* < 0.05; Fig. 3b and Table S3). Gastrointestinal NP accumulation was consistently higher in the SD group than in the MFD and HFD groups at both 7 and 28 days (Fig. 3c, d). Increased dietary fiber intake, regardless of intervention duration, is able to effectively reduce NP accumulation in the GI tract.

On days 7 and 28, NP aggregates were visualized on ileal villi surfaces and in tissue sections of male mice in the SD group (Fig. 3e). By day 28, NIR-NPs penetrated the intestinal epithelium and entered interstitial spaces in the SD group (Fig. 3f), but no NPs were found in the MFD and HFD groups, suggesting that dietary fiber effectively decreased the retention of NPs in intestine villi.

### Intestinal alterations associated with nanoplastic exposure under different dietary interventions

**Fig. 4:**
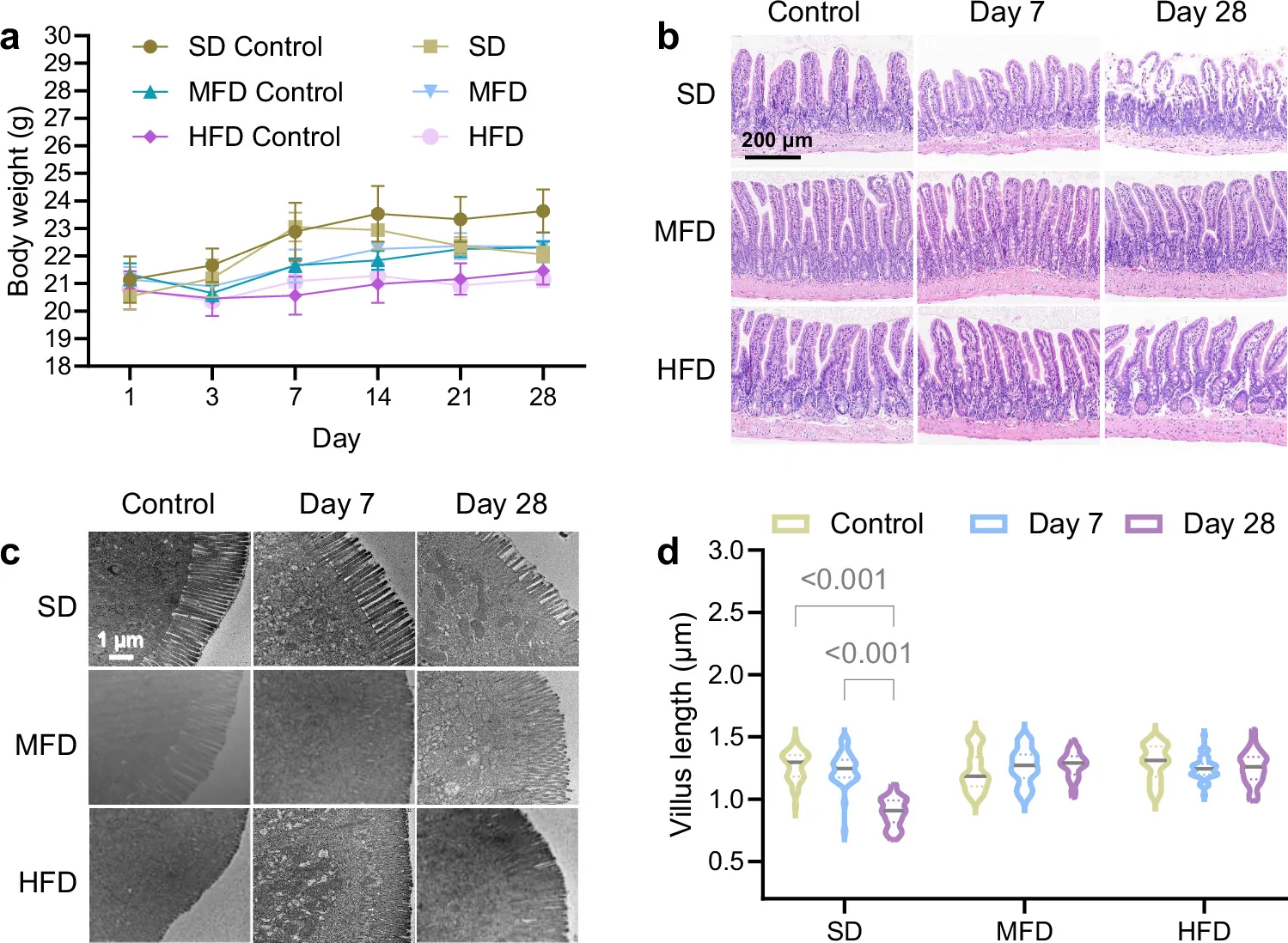
General health status of male mice. **a** Body weight of male mice following 28 days standard diet (SD), medium fiber diet (MFD), and high fiber diet (HFD) interventions. Data are expressed as average ± standard error from six biological replicates; **b** ileal histopathology was evaluated using hematoxylin eosin staining; **c** microscopic structure image of the ileum tissue; representative images are shown from three biologically independent male mice per group with similar results; and **d** length of microvilli in the ileum; microvillus length was measured from multiple villi across three biologically independent male mice per group; significant differences (*p* < 0.05) are indicated above the bars through two-way ANOVA followed by Tukey’s multiple comparison tests. Source data are provided as a Source Data file.

Distinct body weight patterns were observed among different dietary groups due to different dietary interventions during the 28-day exposure. From day 7 to day 28 of exposure, the SD group exhibited a progressive weight reduction compared to the control group (Fig. 4a). This NPs-induced weight loss in non-intervention mice is consistent with the results of a previous study^23^. Both MFD and HFD groups lost weight during the initial exposure period (days 1−3) and then stabilized from day 7 to day 28, mirroring the trend observed in the control group. This pattern may be attributed to enhanced fecal excretion facilitated by increased dietary fiber intake during the initial phase and dietary adaptation afterwards.

**Fig. 5:**
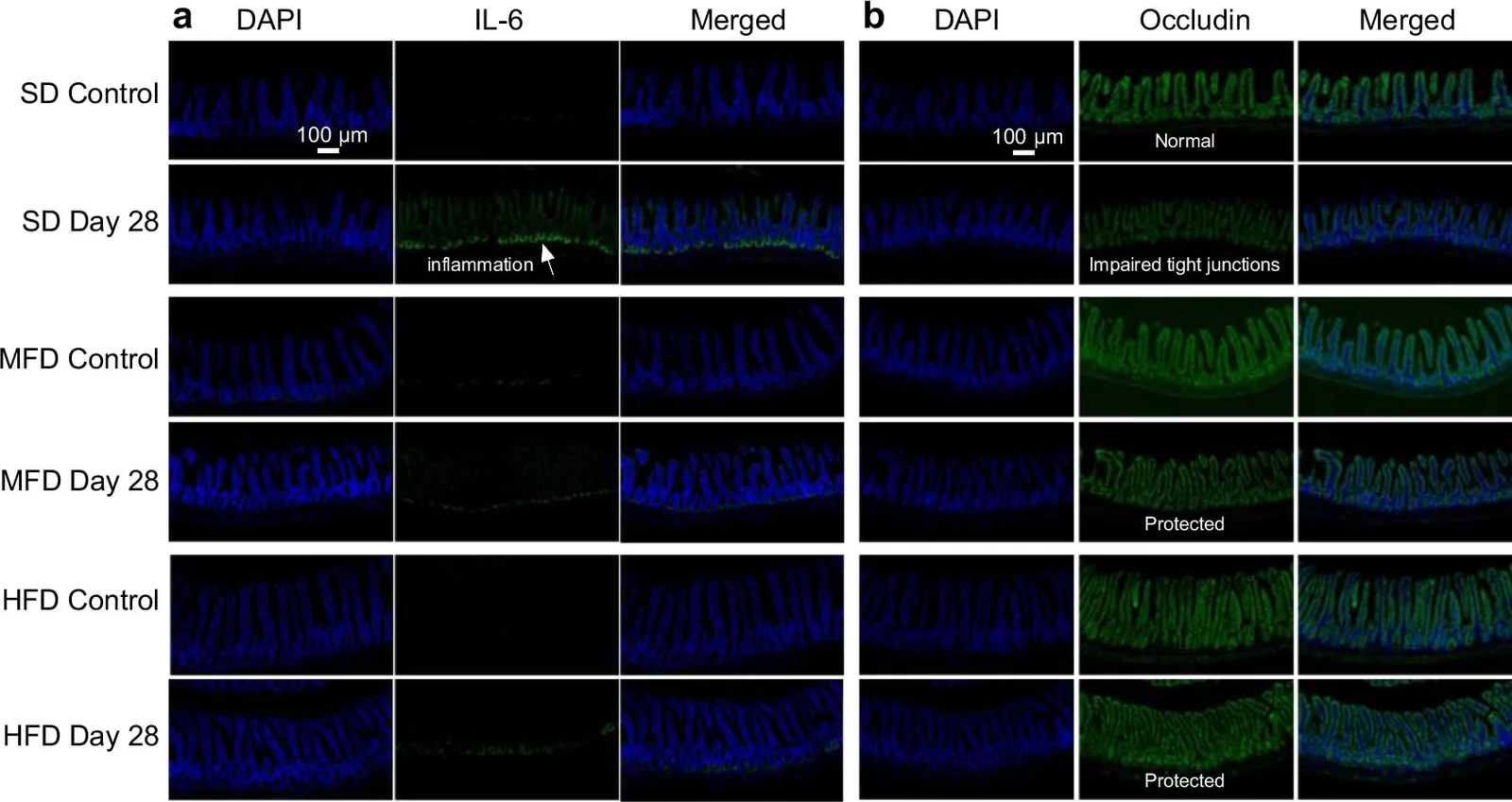
Effects of dietary intervention and nanoplastic exposure on ileal inflammation and intestinal barrier integrity. **a** Immunofluorescence images of IL-6 and **b** occludin in ileal tissues of standard diet (SD), medium fiber diet (MFD), and high fiber diet (HFD) groups at day 28. Representative images are shown from three biologically independent mice per group with similar results.

Histopathological examination at day 7 revealed mild epithelial flattening and crypt loss in the SD group, but no significant morphological alterations in the MFD and HFD groups (Fig. 4b). Ileal epithelial cells were well packed with intact tight junctions and normal microvillus length in all groups at this timepoint (Fig. 4c). By day 28, the SD group developed more severe intestinal damage characterized by marked villous atrophy and extensive crypt loss (Fig. 4b). Disrupted tight junctions and significantly shortened microvilli compared to controls were clearly revealed (*p* < 0.05; Fig. 4c, d), correlating with the persistent weight loss in this group. In contrast, the MFD group maintained intact ileal architecture with well-aligned microvilli, while the HFD group incurred only mild crypt loss and microvillus shortening (Fig. 4d). Long-term exposure to low concentration NPs via food intake induced inflammation (high level IL-6) of small intestinal villus (SD group; Figs. 5a, S2a), which was significantly alleviated by including dietary fiber in diets. Moreover, the decreased occludin expression indicates disruption of intercellular tight junctions and impairment of the intestinal epithelial barrier (Figs. 5b, S2b). Moderate dietary fiber supplementation would alleviate NPs-induced intestinal injury.

### Effects of dietary intervention and nanoplastics exposure on liver health

**Fig. 6:**
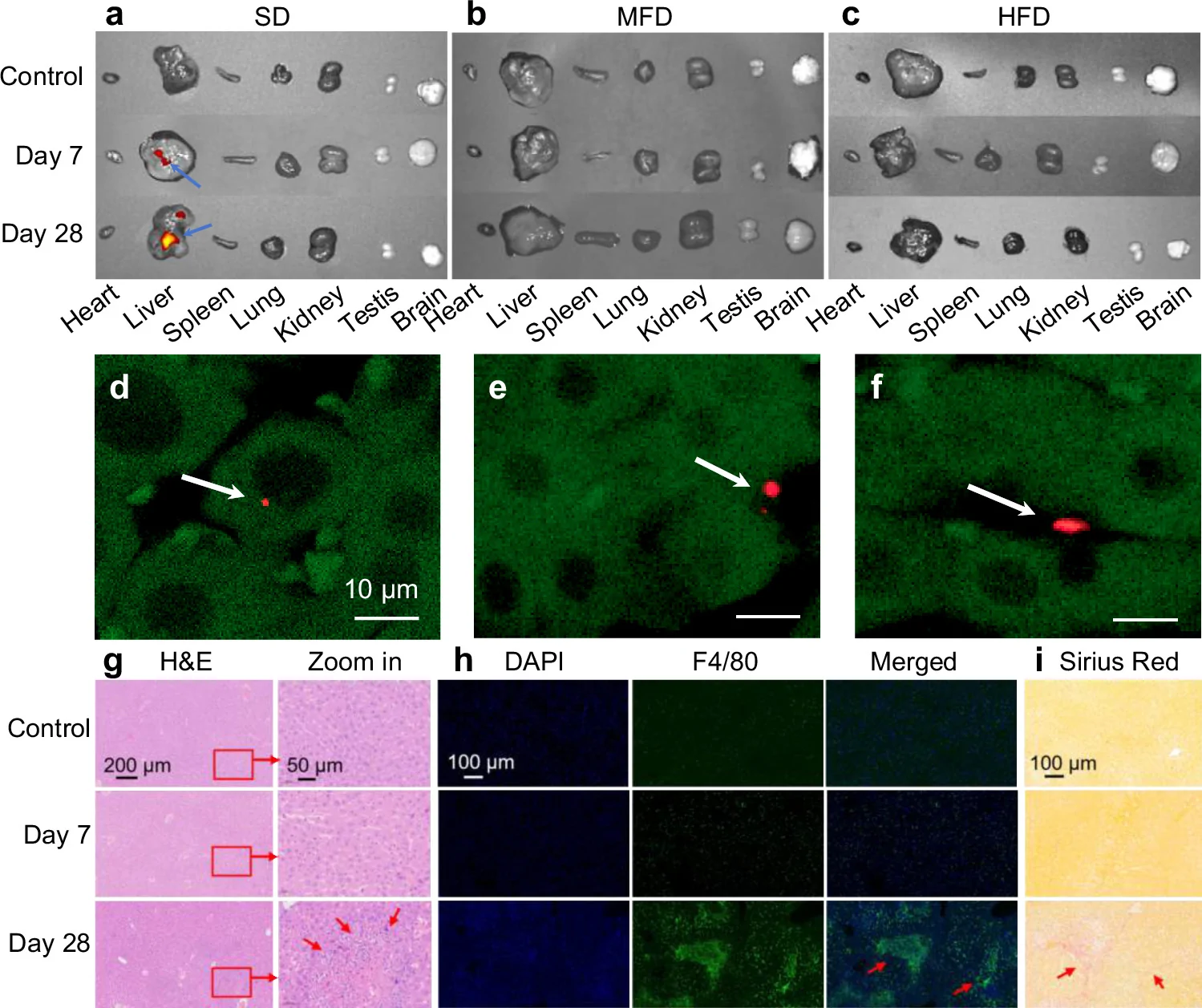
Distribution of near-infrared nanoplastics (NIR-NPs) in important organs and histological assessment of liver tissues. **a**–**c** Fluorescence imaging of NIR-NPs in organs of male mice fed with standard diet (SD), medium fiber diet (MFD), and high fiber diet (HFD), respectively; **d** a NIR-NP single particle within a liver cell; and **e**, **f** NIR-NPs present in the intercellular space of liver; **g** hematoxylin and eosin (H&E) staining of liver tissues from SD mice; **h** immunofluorescence staining of F4/80 in liver tissues from SD mice; and **i** Sirius Red staining of liver tissues from SD mice. Representative images are shown from three biologically independent male mice per group with similar results.

It should be noted that intense NIR fluorescence was detected in mouse liver fed with SD food (3/3) (Fig. 6a), but not with MFD and HFD food (Fig. 6b, c). Importantly, NIR-NPs were found within liver tissue sections (3/3) via fluorescence microscopic imaging (Fig. 6d−f). A single NIR-NP particle was observed within liver cell (Fig. 6d), which may pose long-term threats to liver function. Hematoxylin and eosin staining of liver tissues indicates that the SD group showed evident hepatic inflammatory changes, including focal inflammatory cell infiltration and disrupted local hepatic architecture after 28 days of NP exposure (Fig. 6g). Consistently, F4/80 staining was increased in the SD group, indicating enhanced macrophage infiltration and hepatic immune activation (Fig. 6h). Sirius Red staining also showed limited collagen-positive signals in the SD group, suggesting mild extracellular matrix deposition, although no severe fibrosis was observed (Fig. 6i). Due to the difficulty in detecting trace NPs in biological tissues and organs, we could not rule out the possibility of NPs being present in other organs such as heart, spleen, lung, kidney, testis, and brain. Longer exposure time and more complicated scenarios are required to ratify or reject such possibility.

### Simulated digestion alters cellulose structure and promotes its association with nanoplastics in vitro

**Fig. 7:**
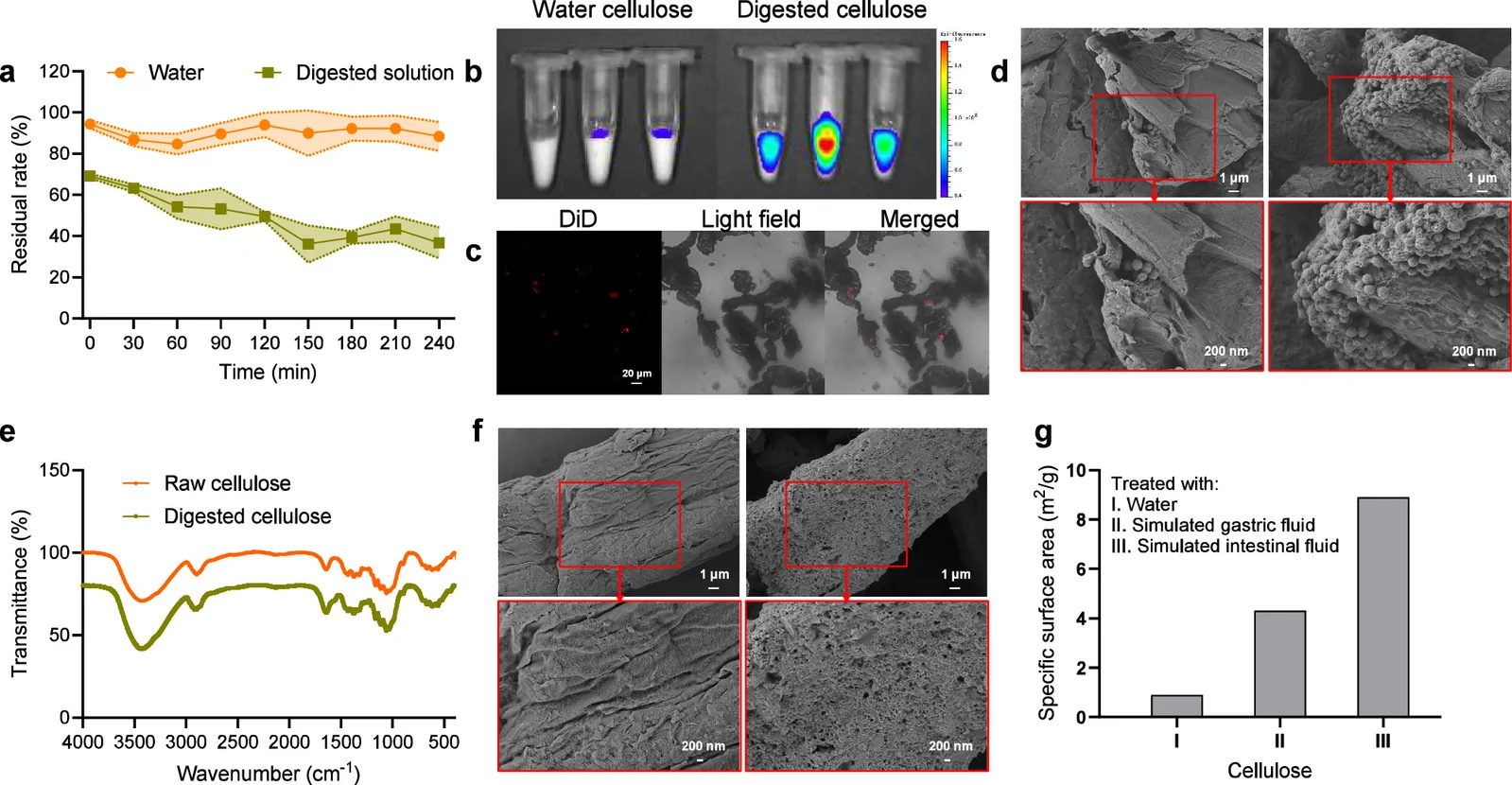
Physical mechanism of dietary fiber in mitigating exposure risk of nanoplastics (NPs). **a** Influence of digestion in vitro on dietary cellulose harvesting NPs; data were presented as average ± standard error from three independent samples; **b** fluorescence images of water-treated and digested cellulose harvesting NPs; **c** confocal fluorescence images and **d** scanning electron microscope (SEM) images of digested cellulose harvesting NPs; **e** Fourier infrared spectroscopy and **f** SEM images of pristine and digested cellulose; **g** influence of digesting methods on the specific surface areas of cellulose. Representative images are shown from three independent samples with similar results. Source data are provided as a Source Data file.

The pristine cellulose, which was added to produce high fiber diet, was used to elucidate the physical mechanism of dietary fiber in mitigating exposure risk of NPs in vitro. The results indicated that the digestion process significantly increased the NPs-harvesting property of cellulose (Fig. 7a). Water-treated cellulose could hardly remove NPs from water while digested cellulose could efficiently remove NPs from water and settle down (Fig. 7b). Interestingly, NPs did not form homogeneous complexes with cellulose, but formed some local high-concentration regions (Fig. 7c). This is further confirmed by SEM images, which show both encapsulation and local adhesion (Fig. 7d). There was no significant change in chemical functional groups (Fig. 7e). Notably, significant morphological change was observed in digested cellulose, which shows more abundant porous microstructures than pristine cellulose (Fig. 7f). The specific surface area of digested cellulose increased up to 10 times that of pristine cellulose (from 0.8898 to 8.9019 m^2^ g^−1^) (Fig. 7g). The dramatically increased surface area and enhanced porous structure might directly result in harvest of NPs by digested fiber, which decreases the bioavailability of NPs.

## Discussion

The present study shows that increasing dietary-fiber content is associated with more rapid fecal elimination and lower gastrointestinal retention of ingested NIR-NPs in male mice, consistent with previous studies indicating that dietary components could influence the gastrointestinal elimination and/or body burden of environmental contaminants. For instance, male and female mice fed with cookies containing 8% wheat bran dietary fiber increased heavy metal excretion by 25% compared to a control group^24^. Dietary fiber intervention was deemed useful in reducing serum levels of perfluoroalkyl substances (i.e., perfluorooctanoic acid, perfluorooctane sulfonate, and perfluorononanoic acid)^21,25^. Beyond fiber, other dietary components also promote NP/MP excretion. Probiotic supplementation (i.e., *Lacticaseibacillus paracasei* DT66 and *Lactiplantibacillus plantarum* DT88) achieved 55% excretion rate for 5 μm MPs compared to 41% for controls^26^. PCC-Chitosan administration improved the human elimination rate of 20–500 μm MPs by 45%^27^. Additionally, bioactive compounds such as cyanidin-3-O-glucoside and quercetin have been shown to facilitate MPs/NPs excretion^28,29^. Compared to current dietary interventions for NP elimination, a high-fiber diet offers superior practical advantages. Dietary fiber is naturally abundant in foods such as corn, potato, fruits (e.g., raspberry, guava, kiwi, etc.), and vegetables (e.g., chickpea, artichoke, celery, etc.), and therefore highly accessible for daily consumption. Dietary fiber is an important dietary factor influencing the fecal elimination and gastrointestinal fate of ingested NPs.

Following oral intake, the first several hours represent a critical window characterized by high gastrointestinal burden of NPs and sustained contact with the intestinal epithelium. Orally ingested NPs subsequently enter systemic circulation through multiple uptake pathways, including epithelial cell endocytosis, transcytosis via microfold (M) cells in Peyer’s patches, and persorption^30,31^. It was demonstrated that PS NPs could pass intestinal wall within 3 h after oral administration and were subsequently captured by liver^32,33^. A tracing study showed that lanthanide-labeled PS particles moved from the stomach and small intestine at 1 h toward the distal gut by 4 h, with systemic organ burden peaking at 4 h^34^, indicating a rapid intestinal uptake and translocation of NPs after ingestion. In the present study, increased dietary fiber intake accelerated fecal elimination of NIR-NPs during the initial excretion window (0–6 h) (Fig. 2e), lowering both total exposure dose and duration. This early clearance may reduce the opportunity for NP translocation across the intestinal barrier by shortening their retention time in critical absorption sites (e.g., small intestine), preventing NPs from interacting with and being taken up by the intestine.

Under repeated exposures, persistent gastrointestinal retention of NPs may prolong particle–mucosa contact, resulting in sustained local exposure and progressive impairment of intestinal barrier integrity^33^. Chronic PS NP exposure has been shown to induce oxidative stress and inflammation, reduce the expression of barrier-associated proteins, including ZO-1, occludin, claudin-1 and MUC-13, and increase intestinal permeability, with some inflammatory and oxidative responses becoming more pronounced with increasing exposure duration^33,35^. Importantly, barrier impairment may not merely result in NP toxicity but also influence subsequent particle uptake and translocation. Disruption of the mucus layer reduces the physical separation between luminal particles and the epithelium, whereas compromised tight junctions increase epithelial accessibility and facilitate trans-barrier passage. Experimental disruption of the intestinal barrier has been shown to enhance the uptake and systemic translocation of PS-NPs, accompanied by increased particle distribution to the liver, lungs, kidneys, heart and brain^36^. These observations support a potentially reinforcing relationship between NP retention and barrier injury, whereby prolonged retention increases mucosal exposure and barrier damage, while impaired barrier integrity may in turn increase the accessibility and translocation of subsequently encountered particles.

In the present study, prolonged NP exposure was accompanied by increased gastrointestinal NP accumulation and progressively aggravated mucosal and barrier injury in the SD group (Figs. 3–5), suggesting that repeated ingestion may sustain intestinal particle exposure over time and contribute to cumulative mucosal injury. In contrast, increasing dietary fiber intake maintained lowered gastrointestinal NP accumulation and attenuated intestinal inflammation and barrier disruption during prolonged exposure. Moreover, prominent hepatic NIR signals and inflammatory alterations were observed after long-term exposure only in the standard-fiber group, whereas corresponding hepatic fluorescence was not detected in the MFD or HFD groups (Fig. 6). Collectively, increasing dietary fiber intake may limit the reinforcing relationship between persistent intestinal NP exposure and barrier injury and reduce the likelihood for subsequent gut-liver axis transport of NPs (Fig. S3).

Dietary fiber may promote NP elimination through a sequestration–transit–elimination process. Our in vitro simulated digestion experiments showed that gastrointestinal processing remodeled cellulose into a more porous structure without appreciable changes in its chemical functional groups. Together with the observed adhesion and encapsulation of NPs, a predominantly physical mechanism may be that digestion-induced structural remodeling enhances particle–fiber association and reduces the fraction of freely dispersed NPs within the intestinal lumen. Due to their resistance to enzymatic degradation in the small intestine, cellulose-bound NPs remain intact during GI transit and are efficiently delivered to the colonic environment^37^. Insoluble dietary fiber mechanically stimulates the large intestine, promotes mucus secretion and water retention, and thereby shortens intestinal transit time^38^ and accelerates fecal excretion of NPs. In the present study, the residual concentration of NIR-NPs in the HFD group 24 h after oral intake was approximately seven times lower than that in the SD group (Fig. 2). Concentrations of NIR-NPs in the GI tract are reduced by substantial excretion, which minimizes contact opportunity or bioavailability with the intestinal epithelium and thereby lowers the risk of NPs-induced intestinal inflammation and NPs biological transport across the epithelial barrier.

The gut microbiota and its metabolites may represent an additional pathway through which dietary fiber modulates the intestinal fate of NPs. Dietary fiber is an important substrate shaping the composition and metabolic activity of the gut microbiota, and microbial fermentation of dietary fiber generates short-chain fatty acids, including acetate, propionate, and butyrate, which contribute to the maintenance of the mucus layer, epithelial barrier function, and mucosal immune homeostasis^39,40^. Conversely, prolonged dietary fiber deprivation can shift microbial substrate utilization toward host-derived mucin glycans, resulting in erosion of the mucus barrier and increased epithelial accessibility^41^. These effects may be particularly relevant to NP exposure because the integrity of the mucus and epithelial barriers determines the accessibility of luminal particles to the intestinal epithelium and their subsequent uptake^36,42^. Polystyrene NPs have also been reported to perturb microbiota–host interactions, and interfere with the host-bacteria signal communication mediated by extracellular vesicles, accompanied by reduced expression of barrier-associated proteins and increased intestinal permeability^33^. Thus, dietary fiber-induced modulation of the gut microbiota and its metabolic environment may further reduce sustained mucosal exposure to NPs by supporting mucus–epithelial barrier homeostasis and gastrointestinal physiological function.

Increased consumption of dietary fiber was reported to ameliorate certain intestinal disorder. For instance, cellulose and lignin, due to their low fermentability and high water-holding capacity, significantly increase fecal bulk, accelerate colonic transit, dilute potential carcinogens, and reduce intraluminal pressure, thereby lowering the risk of diverticular disease and colorectal cancer^16^. Insoluble fiber from wheat bran has been found to effectively restore intestinal barrier function^43^. Additionally, colonic microbiota ferment dietary fiber to produce short-chain fatty acids, which enhances the differentiation of immune cells and the production of the anti-inflammatory cytokine IL-10, and thus alleviates intestinal inflammation^37^. A person is estimated to ingest approximately 68,000 pieces of NPs annually through food, and increasing NPs concentrations are projected to pose significant health risks^44^. Insoluble dietary fiber may mitigate these effects and concurrently alleviate inflammatory bowel disease.

Daily intake of dietary fiber is suggested to be 25−38 g per person^15,45^, which unfortunately is not satisfied in most countries across the globe. For example, the dietary fiber intakes by adults in China, the United States, and European countries are approximately 17^46^, 16.2^15^, and 16–24^43^g per day. Even worse, takeout fast food, which has become increasingly popular, is often packed with plastic containers that may release large quantities of MPs and NPs. People who order takeout food 4−7 times a week may ingest 12−203 pieces of MPs through plastic containers^47^. The annual sales by Meituan, the biggest takeout delivery company in China, increased by approximately three times within the recent five years, reaching 365 billion RMB in 2025 (Fig. S4). Against this backdrop, it is important to determine whether dietary factors can influence the gastrointestinal fate and biological effects of ingested NPs. The present study provides preclinical evidence that dietary-fiber content can influence NP elimination and intestinal exposure in male mice, thereby supporting further investigation of diet–NP interactions under physiologically relevant conditions.

The present study assessed tissue-associated NPs primarily using NIR fluorescence. Although this approach enabled sensitive spatiotemporal tracking, complementary analytical methods, such as pyrolysis-GC/MS, Raman spectroscopy, Fourier-transform infrared spectroscopy, or stable-isotope-based quantification, would provide independent confirmation of the chemical identity and tissue burden of NPs. Direct evidence for cellulose–NP interactions was derived primarily from in vitro simulated digestion experiments, and the extent to which such interactions occur in vivo, as well as their relative contribution to overall NP clearance, remains to be established. In addition, modulation of the gut microbiota by dietary fiber may constitute a potential mechanism affecting NP clearance and reduced intestinal exposure, requiring further investigation. Finally, the present study was conducted exclusively with male mice, and the dietary-fiber levels used, particularly the 50% fiber diet, substantially exceed those typical of human diets. The high-fiber condition should therefore be regarded as a proof-of-concept intervention rather than a directly translatable dietary regimen. Although the 15% fiber diet represents a more moderate intervention, it likewise cannot be directly translated into a human dietary recommendation. Further studies are needed to incorporate both sexes, physiologically relevant fiber levels, and ultimately human populations before these findings can be translated into dietary recommendations or public-health strategies.

## Methods

### Materials

Sodium dodecyl sulfate (SDS; 92.5–100.5%), styrene (C_8_H_8_, 99%), acrylic acid (CH_2_ = CHCOOH, 99%), and divinylbenzene (C_10_H_10_, 55% mixture of isomers) were purchased from Shanghai Macklin Biochemical (Shanghai, China). Sodium persulfate (Na_2_S_2_O_8_, 98%) was obtained from Tianjin Damao Chemical Reagent (Tianjin, China). Near-infrared fluorescent dyes DiD (DiIC18 (5)) (λEx/λEm: 644/663 nm) and DiR (DiIC18 (7)) (λEx/λEm: 748/780 nm) were purchased from Abbkine Scientific (Wuhan, China).

### Preparation and characterization of near-infrared fluorescence nanoplastics

Specifically, SDS (0.05 g) was dissolved in 50 mL ultrapure water. A monomer mixture comprising styrene (5 mL), acrylic acid (0.25 mL), and divinylbenzene (0.25 mL) was added to the SDS solution. Polymerization was initiated with sodium persulfate and conducted at 65 °C under continuous stirring for 5 h, yielding original polystyrene NPs (300 ± 4.5 nm). Original NPs were then aged by exposure to ultraviolet irradiation (λ = 254 nm) for 15 days to obtain the aged NPs. Aged NPs exhibited spherical morphologies, with sizes of 290 ± 6.9 nm and zeta potentials of –10 ± 1.1 mV. Fourier-transform infrared spectroscopy analysis revealed characteristic stretching vibrations for C = O (1709 cm^–1^), C–OH (1397 cm^–1^), and C–O (1031 cm^–1^) in aged NPs and NIR NPs (Fig. S5), consistent with oxygen-containing functional groups reported for environmentally aged plastic particles.

Fluorescent dyes DiD (DiIC18 (5)) and DiR (DiIC18 (7)) were encapsulated into aged NPs in a specific ratio via a swelling method, generating NIR-NPs. The microstructures of the as-prepared NIR-NPs were visualized by scanning electron microscope (S-4800, Hitachi, Tokyo, Japan) and transmission electron microscopy (JEM-1400Plus, Tokyo, Japan). Particle size and zeta potential were measured with Zetasizer Nano S90 (Malvern, UK). Functional groups on the surfaces of NPs were characterized with a Fourier-transform infrared spectrometer (EQUINOX 55; Bruker, Germany). Photoluminescence spectra were acquired with a fluorescence spectrometer (FS5, Edinburgh Instruments, Livingston, UK). Fluorescence signals of NPs were processed with an optical imaging system (UVP Chemstudio touch, Analytik Jena; Jena, Germany).

### Animal experiments

Male SPF BALB/c nude mice (6 weeks old) were purchased from Guangdong Zhiyuan Biomedical Technology (Guangzhou, China). Upon arrival, animals underwent acclimation for one week under controlled conditions: temperatures at 23–26 °C, relative humidity of 60–70%, and a 12-h light/dark cycle (lights on 8:00 AM to 8:00 PM). Male mice had free access to food and sterilized water. As the present study did not investigate a sex-specific disease or condition, sex was not included as an experimental variable in the experimental design and/or data analysis. All procedures were approved by the Ethics Committee of Guangzhou Medical University (Approval No. GY2024-630).

Seventy-two male mice were randomly assigned to six dietary intervention groups (*n* = 12 each), including three short-term (7 days) and three long-term (28 days) groups. Each group received one of the following diets: SD containing 5% cellulose (Table S4), MFD with 15% cellulose (Table S5), and HFD with 50% cellulose (Table S6). Within each dietary group, male mice were further divided into exposure (NP-exposed; *n* = 6) and control (NP-free; *n* = 6) subgroups. The 5% cellulose level in the SD is aligned with the established standard for purified rodent diets, corresponding to approximately 12 g per 1000 calories^15,46^. In comparison, the 15% (MFD) and 50% (HFD) cellulose levels equate to approximately 42 and 240 g per 1000 calories, respectively. The SD and MFD represent low and moderate fiber intake conditions, whereas the HFD is included as a reference to provide proof-of-concept evidence.

A repeated oral gavage protocol of NIR-NPs administration (1 μg g^−1^ day^−1^) conducted between 8:00–9:00 daily was implemented^44,48^. Throughout the exposure period, male mice were regularly monitored for general appearance, activity, food and water intake, body weight, and signs of pain or distress. Animals showing severe or persistent distress were subject to predefined humane endpoints in accordance with the approved animal protocol. At the designated experimental endpoints, male mice were anesthetized with isoflurane (3–4% for induction and 1–2% for maintenance) and subsequently euthanized by cervical dislocation under anesthesia before tissue collection. Tissues from three male mice per group were homogenized for NPs quantification, while the remaining three male mice were subject to histological analysis. Intestinal contents were reserved for residual NP analysis.

### Fluorescence imaging in vivo and in vitro

At 0, 6, 12, and 24 h post-gavage on days 7 and 28, six male mice per group underwent isoflurane anesthesia, followed by in vivo fluorescence imaging using the IVIS Spectrum system (PerkinElmer; Waltham, MA, USA). Mouse feces were collected concurrently during 0–6, 6–12, and 12–24 h intervals on the same days for in vitro fluorescence imaging. Terminal in vitro imaging of GI tracts and organs was also performed after 7 and 28 d exposure to visualize NPs fluorescent signals.

### Quantification of nanoplastics in feces and gut tissues

Feces samples underwent freeze drying, weighing, and ultrasonic homogenization in 10-mL 75% ethanol. Gastrointestinal tract tissue samples were weighed and digested with 10% potassium hydroxide (KOH; 10:1 volume to weight), and incubated at 50 °C for 24 h with 180 rpm shaking^44^. Tissue digestates were diluted to 5 mL with deionized water.

Fluorescence analysis on fecal homogenates and tissue digestates was performed using the IVIS optical imaging system. To mitigate matrix interferences, NPs concentrations were determined with a standard addition methodology, which corrects for signal suppression or enhancement by matching the sample matrix^49^. Homogenates were partitioned into five or six aliquots, with four or five aliquots spiked with NP standards at varying concentrations to generate calibration curves. The absolute values of *x*-axis intercepts of these curves represent the NP concentrations in the target samples^49^. The excretion (*η*_feces_) and intestinal accumulation (*η*_intestine_) rates of NPs are expressed as

1 $${\eta }_{{{\mathrm{feces}}}}={M}_{{{\mathrm{feces}}}}/{M}_{{{\mathrm{intake}}}}\times 100\%$$

2 $${\eta }_{{{\mathrm{intestine}}}}={M}_{{{\mathrm{intestine}}}}/{M}_{{{\mathrm{intake}}}}\times 100\%\,$$

where *M*_feces_, *M*_intake_, and *M*_intestine_ represent cumulative NP mass in feces, total administered NP amount, and residual NP amount in GI tracts, respectively. Concentrations of NPs in fecal and gastrointestinal samples from all dietary groups were quantified using matrix-matched fluorescence calibration curves. Representative standard curves for each dietary group and sample matrix are provided in Fig. S6. Future studies combining NIR tracking with other complementary techniques such as pyrolysis-GC/MS, Raman spectroscopy or Fourier transform infrared spectroscopy would be particularly valuable for further confirming intact NPs in tissues.

### Intestine and liver histology

Ileum and liver tissues were fixed in 4% paraformaldehyde for 24 h, embedded in paraffin blocks, and serially sectioned at a thickness of 6 μm. Hematoxylin and eosin staining was performed according to standard protocols. Briefly, tissue sections underwent deparaffinization and rehydration, followed by hematoxylin staining to visualize nuclei. After differentiation and bluing, eosin counterstaining was applied to highlight cytoplasmic and extracellular components. Finally, the sections were dehydrated, cleared, and mounted for microscopic examination (Leica Aperio CS2, Germany).

For immunofluorescence staining, the paraffin-embedded ileal and liver sections were deparaffinized, rehydrated, and subjected to antigen retrieval. After being treated with a blocking buffer to reduce non-specific binding, ileum sections were incubated overnight at 4 °C with an anti-IL-6 polyclonal antibody or an anti-occludin polyclonal antibody (Servicebio, China; 1:500 dilution), and liver sections were incubated with an anti-F4/80 polyclonal antibody (Servicebio, China; 1:500 dilution). The sections were washed and incubated with an Alexa Fluor 488 labeled goat anti-rabbit IgG secondary antibody (1:400 dilution). Finally, the sections were incubated with 4′,6-diamidino-2-phenylindole (DAPI) for 10 min at room temperature. Fluorescence images were obtained using a fluorescence microscope, and the fluorescence intensity was quantitatively analyzed using ImageJ.

For Sirius Red staining, paraffin-embedded liver tissue sections were dewaxed, rehydrated, and rinsed with distilled water. The sections were stained with Sirius Red solution A at 65 °C for 30 min, rinsed with ultrapure water, stained with modified Sirius Red solution B for 2 min, rinsed again, and then stained with modified Sirius Red solution C for 1 h. After staining, the sections were dehydrated rapidly in anhydrous ethanol, cleared in xylene, and mounted with neutral resin. The sections were photographed using a light microscope (Leica Aperio CS2, Germany).

Ileum and liver samples were fixed in 4% paraformaldehyde/phosphate buffer solution (PBS), embedded in a frozen section embedding medium, and flash-frozen on dry ice. Subsequently, 5–8 μm sections were prepared using cryostat sectioning. Tissue sections were incubated with fluorescein isothiocyanate phalloidin solution for 30 min. Following three PBS washes, the sections were mounted using anti-fluorescence quenching mounting medium. Images were acquired using a laser confocal microscope (Zeiss 900; Jena, Germany).

### Transmission electron microscope analysis of intestine

Intestinal samples were fixed in 2.5% glutaraldehyde solution at 4 °C. After three PBS washes, specimens were post fixed in 1% osmium tetroxide (2–3 h) and stored at 4 °C. Following dehydration and embedding, ultrathin sections (50–70 nm) were prepared with an ultramicrotome (Leica EM UC7, Leica Microsystem, Germany), stained with 3% uranyl acetate and lead citrate, and examined by transmission electron microscopy (JEM-1400Plus; Tokyo, Japan).

### In vitro simulated digestion

Simulated gastric fluid (pH 2.5) was prepared by dissolving potassium chloride (0.51 g L^−1^), sodium bicarbonate (2.1 g L^−1^), sodium chloride (2.8 g L^−1^), potassium dihydrogen phosphate (0.12 g L^−1^), magnesium chloride hexahydrate (0.020 g L^−1^), and calcium chloride dihydrate (0.022 g L^−1^). Pepsin was added at a final concentration of 0.8 g L^−1^ immediately before use. Simulated intestinal fluid (pH 7.0) was prepared by adding bile salts (1.78 g L^−1^) and pancreatin (0.5 g L^−1^) to the gastric digestion mixture.

All simulated digestive fluids were freshly prepared and pre-warmed to 37 °C before use. For in vitro digestion, cellulose samples were first mixed with simulated gastric fluid and incubated at 37 °C with shaking at 120 rpm for 2 h. After gastric digestion, the pH of gastric fluid was adjusted to 7.0 using sodium bicarbonate solution, and simulated intestinal fluid was added to the mixture. Then the mixture was incubated at 37 °C for another 2 h to simulate small intestinal digestion. For control groups, cellulose samples were incubated in deionized water with the same temperature, shaking speed, and incubation time. Fluorescent NPs were added to the digestive fluid or deionized water before digestion and incubated for the indicated time. After incubation, samples were then separated into supernatant and cellulose-associated precipitates for fluorescence quantification, Fourier infrared spectroscopy, Brunauer Emmett Teller, and imaging analyses.

### Statistics and reproducibility

For in vivo experiments, male mice were assigned to the indicated dietary groups, with no fewer than six individuals per group. In vitro assays, including quantification of fluorescence intensity and NPs concentration, electron microscopy imaging, confocal microscopy imaging, and histopathological assessment were performed using at least three independent biological samples.

The data graphing and statistical analysis were conducted using GraphPad Prism (version 11.0.0, GraphPad Software, USA) and Origin 8.5. All data are expressed as average ± standard error. One-way and/or two-way analysis of variance followed by Tukey’s multiple comparisons test was employed for data comparison, with *p* < 0.05 considered statistically significant.

### Reporting summary

Further information on research design is available in the Nature Portfolio Reporting Summary linked to this article.

## Supplementary information

Supplementary Information

Reporting Summary

Transparent Peer Review file

## Source data

Source Data

## Data Availability

All data supporting the findings described in this manuscript are available in the article, in the Supplementary Materials, and in the source data file. Source data are provided with this paper.
