# Supplementary material for: High fiber diet mitigates exposure risk of nanoplastics in male mice
Source: Nat Commun. 2026 Oct 15;17:11630. doi: 10.1038/s41467-026-78675-7 (PMC13754771; doi:10.1038/s41467-026-78675-7)
Supplement: Supplementary file 1 [file 41467_2026_78675_MOESM1_ESM.pdf]

Supplementary Materials for

High fiber diet mitigates exposure risk of nanoplastics in male mice

Zimin Yu[1,2#], Gulizhabaier Abulipizi[1#], Yanlun Fang[1], Chaomin Qu[1], Leping Ding[1], Ziang Zong[1], Jie Huang[1], Ziyang Chen[1], Juanjuan Zhou[1*], Zhanjun Li[1*], Eddy Y. Zeng[3*]

[1] *School of Biomedical Engineering, Guangzhou Medical University, Guangzhou 511436, China*

[2] *School of Public Health, Guangdong Medical University, Dongguan 523808, China*

[3] *Institute of Environmental Health, School of Environment and Energy, South China University of Technology, Guangzhou 510006, China*

[#] These authors contributed equally: Zimin Yu and Gulizhabaier Abulipizi

* Corresponding authors: Email: jjzhou417@163.com (JZ); Email: zhanjunli@gzhmu.edu.cn (ZL); Email: eddyzeng@scut.edu.cn (EYZ)

This PDF file includes

Figures S1 to S6

Tables S1 to S6

Supplementary Figures

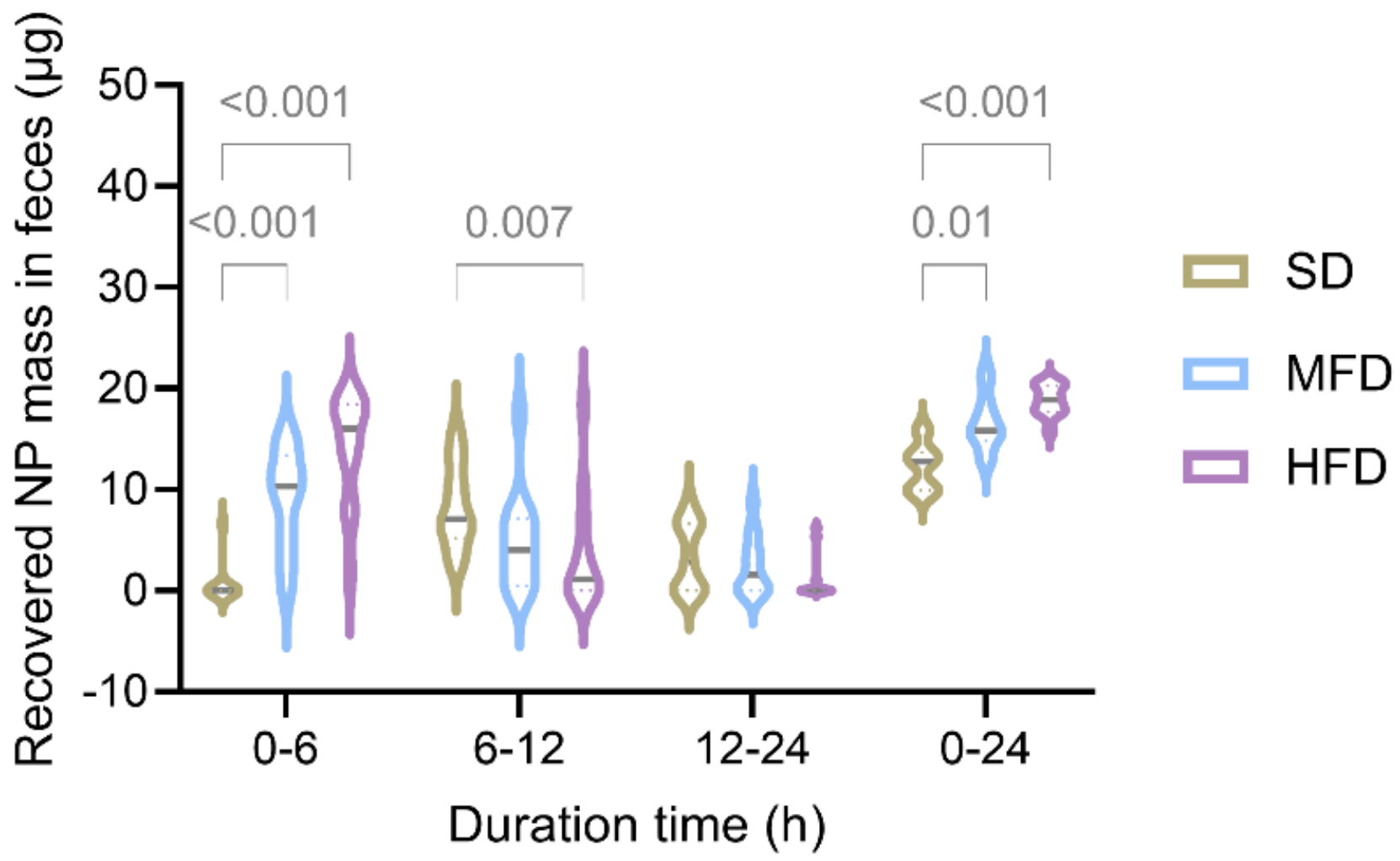

Figure S1. Concentrations of nanoplastics (NPs) in feces after receiving standard fiber (SD), medium fiber (MFD), and high fiber (HFD) diet with 24 h. Statistical analysis was performed through two-way ANOVA followed by Tukey's multiple comparison tests ($n = 6$ biological replicates). Source data are provided as a Source Data file.

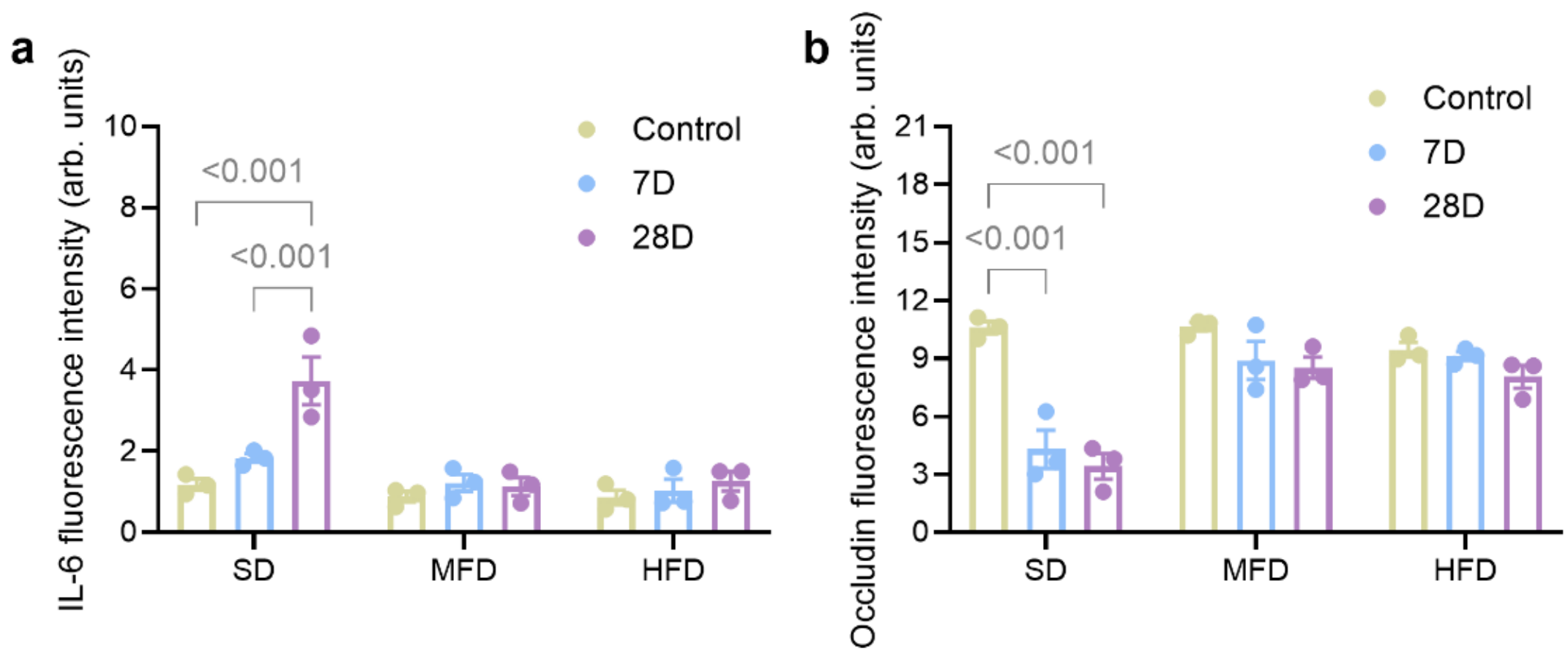

Figure S2. Immunofluorescence staining of IL-6 and occluding in ileal tissues of standard fiber diet (SD), medium fiber diet (MFD), and high fiber diet (HFD) interventions for 7 days (7D) and 28 days (28D): (a) Quantification of IL-6 fluorescence intensity and (b) quantification of occludin fluorescence intensity. Data are expressed as average ± standard error from three biological replicates; significant differences ($p < 0.05$) were performed through two-way ANOVA followed by Tukey's multiple comparison tests. Source data are provided as a Source Data file.

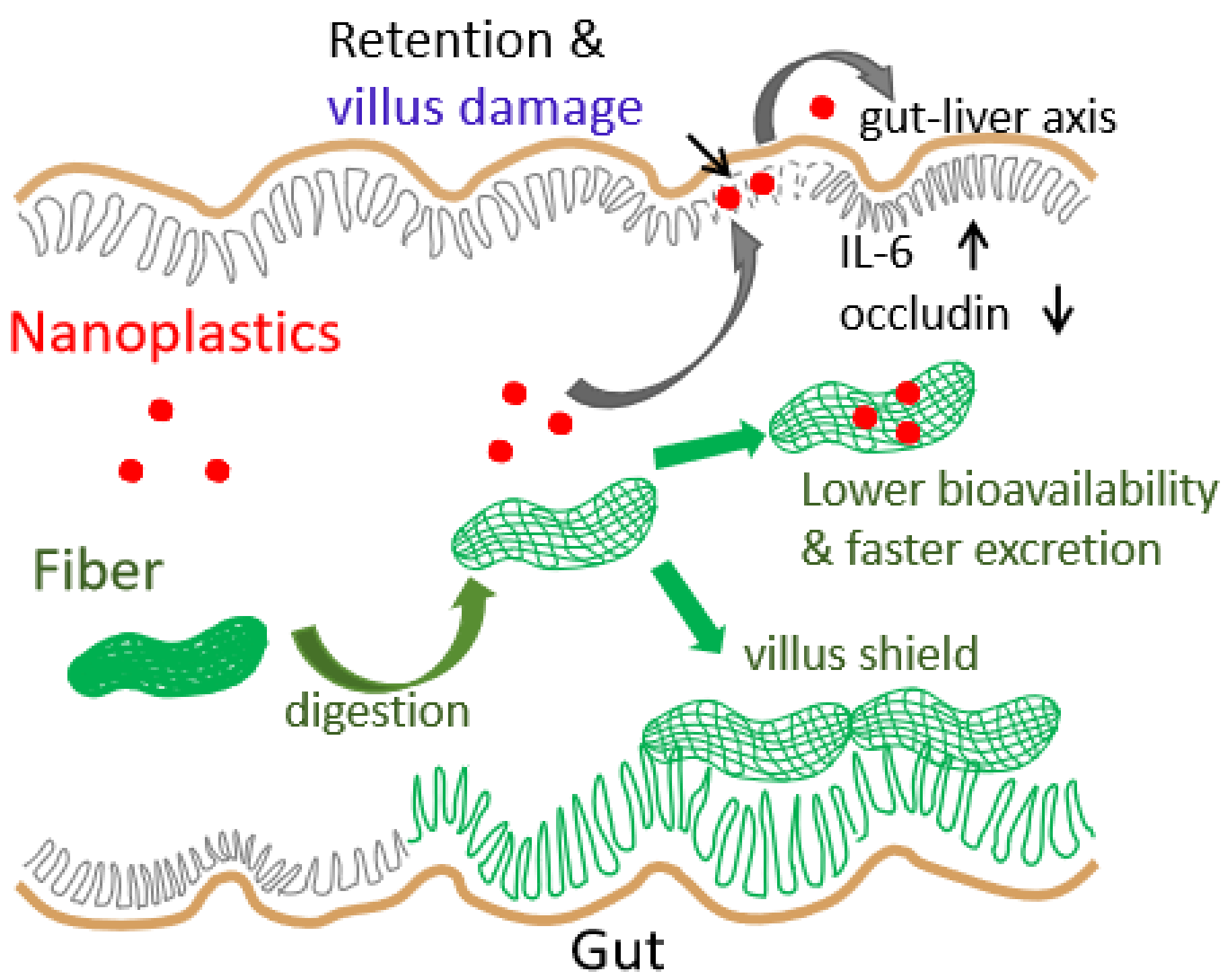

Figure S3. Proposed mechanism of high fiber diet mitigating exposure risk of nanoplastics.

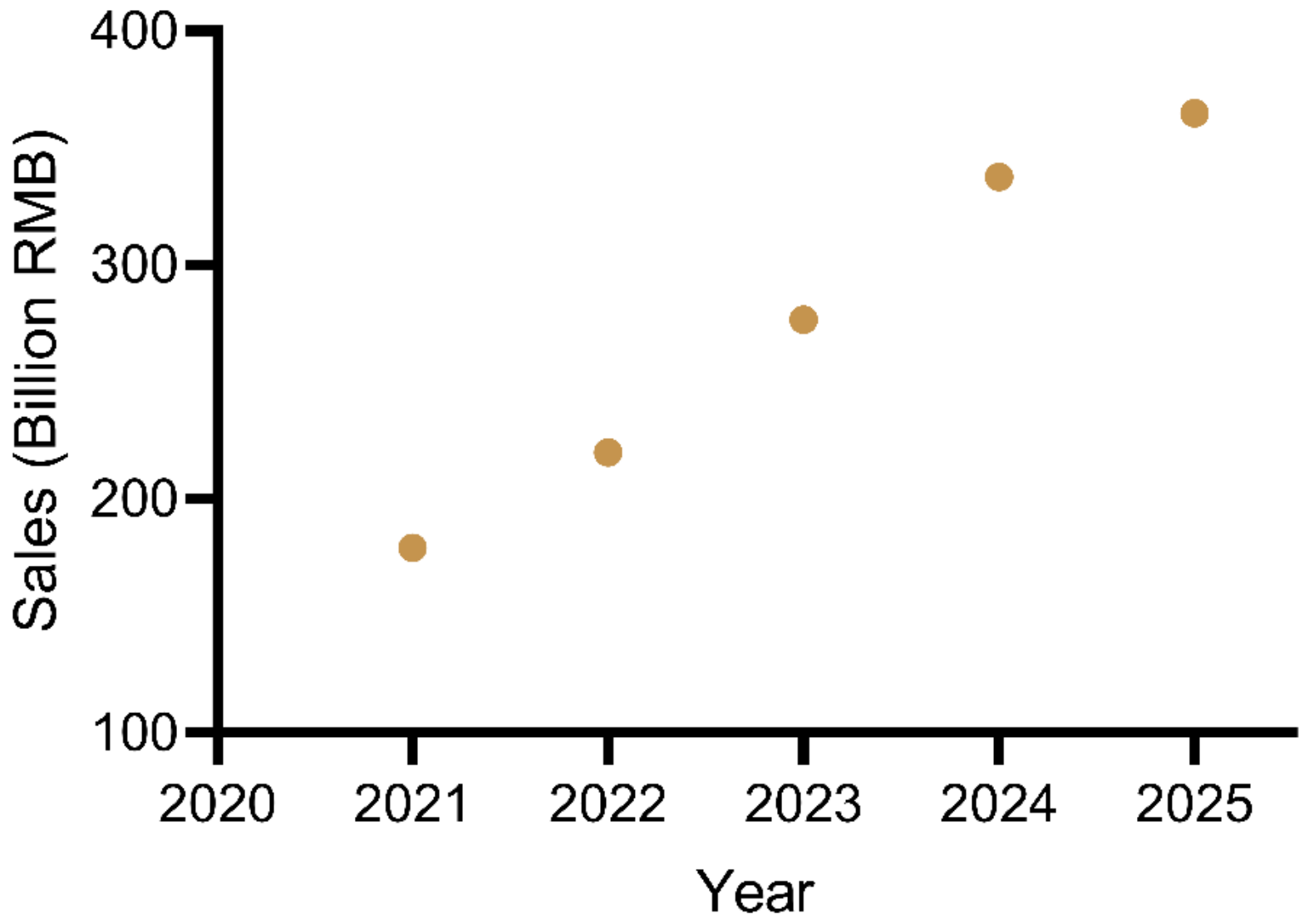

Figure S4. Sales of the largest takeout listed company in China, Meituan, according to its annual financial statements. Source data are provided as a Source Data file.

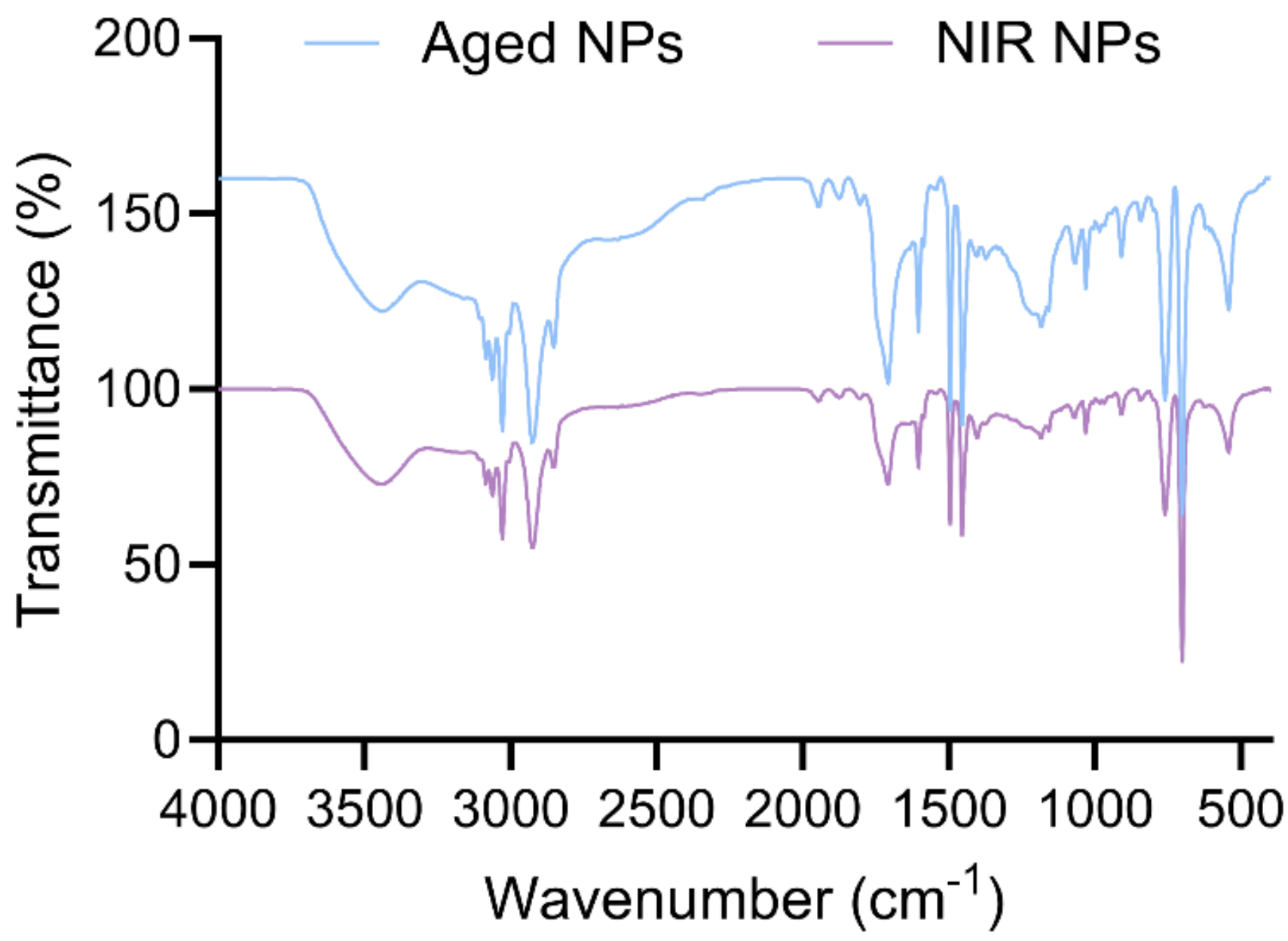

Figure S5. Fourier-transform infrared spectroscopy of aged nanoplastics (Aged NPs) and aged near-infrared nanoplatics (NIR NPs). Source data are provided as a Source Data file.

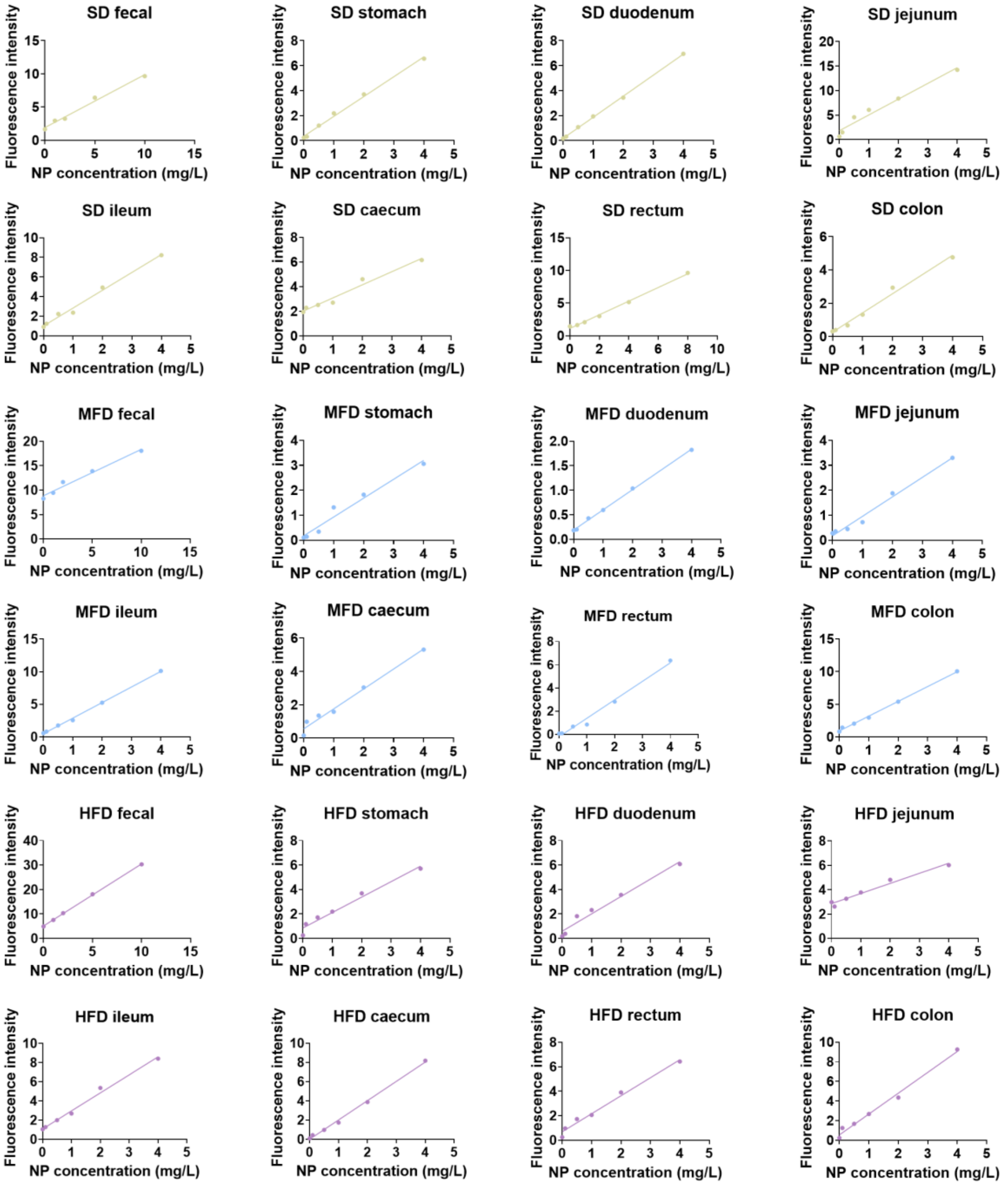

Figure S6. Representative standard curves of fluorescence intensity of aged near-infrared nanoplatics (NIR NPs) in fecal and intestine of standard fiber diet (SD), medium fiber diet (MFD), and high fiber diet (HFD) groups. Source data are provided as a Source Data file.

Supplementary Tables

Table S1. Normalized fluorescence intensity (average ± standard error) of nanoplastics in mice with daily digestion time.

	Standard diet	Medium fiber diet	High fiber diet
0 h	1.00 ± 0.00	1.00 ± 0.00	1.00 ± 0.00
6 h	0.65 ± 0.07	0.46 ± 0.06	0.23 ± 0.04
12 h	0.50 ± 0.12	0.27 ± 0.05	0.13 ± 0.04
24 h	0.22 ± 0.04	0.05 ± 0.02	0.01 ± 0.01

Table S2. Excretion rate (average ± standard error, %) of nanoplastics in mice with daily digestion time.

	Standard diet	Medium fiber diet	High fiber diet
0–6 h	2.8 ± 2.6	46 ± 6.9	71 ± 7.6
6–12 h	42 ± 5.8	25 ± 6.8	18 ± 7.8
12–24 h	17 ± 4.7	12 ± 4.1	5.2 ± 3.1
0–24 h	61 ± 3.2	83 ± 3.8	94 ± 2.1

Table S3. Bioaccumulation rate (average ± standard error, %) of nanoplastics in mice with daily digestion time.

	Standard diet	Medium fiber diet	High fiber diet
Day 7	9.1 ± 0.85	2.0 ± 0.52	1.4 ± 0.26
Day 28	4.8 ± 0.51	1.9 ± 0.27	0.84 ± 0.17

Table S4. Feed formula of AIN-93G Purified Rodent Diet (standard diet).

Ingredient	Heat (kcal g^{-1})	Mass (gram)	Calorie (kcal)
Caisein	4	200	800
L-Cystine	4	3	12
Sucrose	4	100	400
Cornstarch	4	397.5	1590
Dyetrose	4	132	528
Soybean Oil	9	70	630
Cellulose	0	50	0
Mineral Mix #210025	0	35	0
Vitamin Mix #310025	4	10	40
Choline Bitartrate	0	2.5	0

Table S5. Feed formula of AIN-93G Purified Rodent Diet with 15% cellulose (medium fiber diet).

Ingredient	Heat (kcal g^{-1})	Mass (gram)	Calorie (kcal)
Caisein	4	200	800
L-Cystine	4	3	12
Sucrose	4	100	400
Cornstarch	4	397.5	1590
Dyetrose	4	132	528
Soybean Oil	9	70	630
Cellulose	0	167.65	0
Mineral Mix #210025	0	35	0
Vitamin Mix #310025	4	10	40
Choline Bitartrate	0	2.5	0

Table S6. Feed formula of AIN-93G Purified Rodent Diet with 50% cellulose (high fiber diet).

Ingredient	Heat (kcal g^{-1})	Mass (gram)	Calorie (kcal)
Caisein	4	200	800
L-Cystine	4	3	12
Sucrose	4	100	400
Cornstarch	4	397.5	1590
Dyetrose	4	132	528
Soybean Oil	9	70	630
Cellulose	0	950	0
Mineral Mix #210025	0	35	0
Vitamin Mix #310025	4	10	40
Choline Bitartrate	0	2.5	0
