# Supplementary material for: High fiber diet mitigates exposure risk of nanoplastics in male mice
Source: Nat Commun. 2026 Oct 15;17:11630. doi: 10.1038/s41467-026-78675-7 (PMC13754771; doi:10.1038/s41467-026-78675-7)
Supplement: Supplementary file 2 [file 41467_2026_78675_MOESM2_ESM.pdf]

nature portfolio

Corresponding author(s): Eddy Y. Zeng, Zhanjun Li, Juanjuan Zhou

Last updated by author(s): Jul 13, 2026

Reporting Summary

Nature Portfolio wishes to improve the reproducibility of the work that we publish. This form provides structure for consistency and transparency in reporting. For further information on Nature Portfolio policies, see our Editorial Policies and the Editorial Policy Checklist.

Statistics

For all statistical analyses, confirm that the following items are present in the figure legend, table legend, main text, or Methods section.

n/a	Confirmed	
☐	☒	The exact sample size (*n*) for each experimental group/condition, given as a discrete number and unit of measurement
☐	☒	A statement on whether measurements were taken from distinct samples or whether the same sample was measured repeatedly
☐	☒	The statistical test(s) used AND whether they are one- or two-sided<br>*Only common tests should be described solely by name; describe more complex techniques in the Methods section.*
☐	☒	A description of all covariates tested
☐	☒	A description of any assumptions or corrections, such as tests of normality and adjustment for multiple comparisons
☐	☒	A full description of the statistical parameters including central tendency (e.g. means) or other basic estimates (e.g. regression coefficient) AND variation (e.g. standard deviation) or associated estimates of uncertainty (e.g. confidence intervals)
☐	☒	For null hypothesis testing, the test statistic (e.g. *F*, *t*, *r*) with confidence intervals, effect sizes, degrees of freedom and *P* value noted<br>*Give P values as exact values whenever suitable.*
☒	☐	For Bayesian analysis, information on the choice of priors and Markov chain Monte Carlo settings
☒	☐	For hierarchical and complex designs, identification of the appropriate level for tests and full reporting of outcomes
☒	☐	Estimates of effect sizes (e.g. Cohen's *d*, Pearson's *r*), indicating how they were calculated

Our web collection on statistics for biologists contains articles on many of the points above.

Software and code

Policy information about availability of computer code

Data collection	No software was used for data collection.
Data analysis	wps office, GraphPad Prism version 11.0.0, origin8.5, ImageJ, IBM SPSS Statistics (version 22.0) were used for data analysis.

For manuscripts utilizing custom algorithms or software that are central to the research but not yet described in published literature, software must be made available to editors and reviewers. We strongly encourage code deposition in a community repository (e.g. GitHub). See the Nature Portfolio guidelines for submitting code & software for further information.

Data

Policy information about availability of data

All manuscripts must include a data availability statement. This statement should provide the following information, where applicable:

- Accession codes, unique identifiers, or web links for publicly available datasets
- A description of any restrictions on data availability
- For clinical datasets or third party data, please ensure that the statement adheres to our policy

Provide your data availability statement here.

Research involving human participants, their data, or biological material

Policy information about studies with human participants or human data. See also policy information about sex, gender (identity/presentation), and sexual orientation and race, ethnicity and racism.

Reporting on sex and gender	N/A
Reporting on race, ethnicity, or other socially relevant groupings	N/A
Population characteristics	N/A
Recruitment	N/A
Ethics oversight	N/A

Note that full information on the approval of the study protocol must also be provided in the manuscript.

Field-specific reporting

Please select the one below that is the best fit for your research. If you are not sure, read the appropriate sections before making your selection.

☐ Life sciences ☐ Behavioural & social sciences ☒ Ecological, evolutionary & environmental sciences

For a reference copy of the document with all sections, see nature.com/documents/nr-reporting-summary-flat.pdf

Ecological, evolutionary & environmental sciences study design

All studies must disclose on these points even when the disclosure is negative.

Study description	Mice were fed with nanoplastic and dietary fiber to study the influence of fiber content on the bio-transport and excretion of nanoplastics. The main treatment factors were dietary intervention (standard-, medium- and high-fiber diets), nanoplastic exposure (exposed or control) and intervention duration (7 or 28 days, where applicable). The design included comparisons across dietary groups and exposure conditions, with individual mice serving as the experimental units. For quantitative in vivo imaging, excretion and body-weight analyses, six biological replicates were used per exposure or control subgroup. For tissue accumulation and histological analyses, three biologically independent mice per group were analyzed. Statistical analyses considered the relevant treatment factors, and group comparisons were performed using one-way or two-way analysis of variance followed by Tukey's multiple-comparison test, as appropriate.
Research sample	The research sample consisted of male SPF BALB/c nude mice, 6 weeks old, purchased from Guangdong Zhiyuan Biomedical Technology (Guangzhou, China). This mouse model was selected because it is suitable for controlled dietary intervention and in vivo fluorescence imaging of nanoplastic biodistribution and excretion. After acclimation, mice were assigned to standard-, medium- or high-fiber dietary interventions, with or without oral nanoplastic exposure. The sample was intended to represent a controlled mouse model for evaluating dietary modulation of nanoplastic excretion, intestinal exposure and gut-liver axis translocation. No existing datasets were used in this study.
Sampling strategy	Mice were randomly assigned to dietary intervention and exposure groups after one week of acclimation. Samples were collected at predefined time points according to the experimental design, including feces, gastrointestinal contents, ileum and liver tissues. The sample size was selected based on commonly used group sizes in comparable mouse imaging and dietary intervention studies, while ensuring at least three biological replicates for tissue-based analyses and six biological replicates for quantitative in vivo imaging and excretion measurements
Data collection	Body weight and fecal output were recorded manually during the intervention period. In vivo and ex vivo fluorescence signals were acquired using an optical imaging system, fluorescence microscopy and laser confocal microscopy, as appropriate. Histological and ultrastructural images were obtained using light microscopy, fluorescence microscopy, laser confocal microscopy, scanning electron microscopy and transmission electron microscopy. Quantitative fluorescence and image data were analyzed using ImageJ and the corresponding instrument software.
Timing and spatial scale	Data were collected during the predefined 7-day and 28-day animal intervention periods. Body weight was recorded at scheduled time points during the intervention periods, and fecal samples were collected during the indicated post-gavage intervals. In vivo fluorescence imaging was performed at the designated time points after oral nanoplastic administration, and tissue samples were collected at the end of the 7-day or 28-day exposure period. There were no intended gaps between sampling periods within each cohort. The lab study were conducted from 2025-4-1 to 2026-4-17 in guangzhou medical university, guangzhou, China.
Data exclusions	No data were excluded.
Reproducibility	All planned experimental repeats were completed successfully, and no attempts to repeat the experiments failed.

Randomization	Seventy-two mice were randomly assigned to six dietary intervention groups (n = 12 each), including three short-term (7 days) and three long-term (28 days) groups. Each group received one of the following diets: SD containing 5% cellulose, MFD with 15% cellulose, and HFD with 50% cellulose. Within each dietary group, mice were further divided into exposure (NP-exposed; n = 6) and control (NP-free; n = 6) subgroups. The 5% cellulose level in the SD is aligned with the established standard for purified rodent diets, corresponding to approximately 12 g per 1,000 calories. In comparison, the 15% (MFD) and 50% (HFD) cellulose levels equate to approximately 42 and 240 g per 1,000 calories, respectively. The SD and MFD represent low and moderate fiber intake conditions, whereas the HFD was included as a reference to provide proof-of-concept evidence.
Blinding	Personnel responsible for animal feeding, oral exposure and sample collection were not blinded to group allocation because these procedures required knowledge of the dietary and exposure assignments. After sample collection, samples and images were coded, and fluorescence quantification and image analysis were performed without knowledge of group allocation. Group identities were revealed only after data quantification was completed. Statistical analyses were conducted according to a prespecified analysis plan.

Did the study involve field work? ☐ Yes ☒ No

Reporting for specific materials, systems and methods

We require information from authors about some types of materials, experimental systems and methods used in many studies. Here, indicate whether each material, system or method listed is relevant to your study. If you are not sure if a list item applies to your research, read the appropriate section before selecting a response.

Materials & experimental systems

n/a	Involved in the study
☐	☒ Antibodies
☒	☐ Eukaryotic cell lines
☒	☐ Palaeontology and archaeology
☐	☒ Animals and other organisms
☒	☐ Clinical data
☒	☐ Dual use research of concern
☒	☐ Plants

Methods

n/a	Involved in the study
☒	☐ ChIP-seq
☒	☐ Flow cytometry
☒	☐ MRI-based neuroimaging

Antibodies

Antibodies used	anti-IL-6 polyclonal antibody; anti-occludin polyclonal antibody; anti-F4/80 polyclonal antibody; Alexa Fluor 488 labeled goat anti-rabbit IgG secondary antibody
Validation	The primary antibodies used in the present study were commercially available antibodies from Servicebio and were applied according to the manufacturer's instructions for mouse tissue immunofluorescence staining. The anti-IL-6 antibody was validated for immunofluorescence detection in mouse ileal tissue in the present study, showing low background staining in control samples and increased signal in nanoplastic-exposed samples. The anti-occludin antibody was validated by its expected epithelial localization pattern in mouse ileal tissue. The anti-F4/80 antibody was validated by its expected macrophage-associated staining pattern in mouse liver tissue. Secondary-antibody-only controls were used to assess non-specific background fluorescence where applicable

Animals and other research organisms

Policy information about studies involving animals; ARRIVE guidelines recommended for reporting animal research, and Sex and Gender in Research

Laboratory animals	SPF BALB/c nude mice (6 weeks old) were purchased from Guangdong Zhiyuan Biomedical Technology (Guangzhou, China).
Wild animals	N/A
Reporting on sex	All mice were male in the present study.
Field-collected samples	N/A
Ethics oversight	All procedures were approved by the Ethics Committee of Guangzhou Medical University (Approval No. GY2024-630).

Note that full information on the approval of the study protocol must also be provided in the manuscript.

Plants

Seed stocks	N/A
Novel plant genotypes	N/A
Authentication	N/A
