# Supplementary material for: High fiber diet mitigates exposure risk of nanoplastics in male mice
Source: Nat Commun. 2026 Oct 15;17:11630. doi: 10.1038/s41467-026-78675-7 (PMC13754771; doi:10.1038/s41467-026-78675-7)
Supplement: Supplementary file 3 [file 41467_2026_78675_MOESM3_ESM.pdf]

nature portfolio

Peer Review File

High fiber diet mitigates exposure risk of nanoplastics in male mice

Corresponding Author: Professor Eddy Zeng

This file contains all reviewer reports in order by version, followed by all author rebuttals in order by version.

Version 0:

Reviewer comments:

Reviewer #1

(Remarks to the Author)
I do not agree that the authors have demonstrated any important or novel findings in this study. In fact, no mechanistic insights were discussed or elucidated throughout the manuscript. Additionally, several issues require clarification:

Although the authors quantified NP accumulation in various organs, they did not analyze NP levels in blood or feces during the treatment period. Without these data, it is difficult to determine whether the dietary intervention truly promoted NP excretion.

In Figure S4, fecal NP levels were measured only in the standard diet group. The authors should explain why no measurements were conducted for the other treatment groups.

Is it possible that the fiber diet may physically entrap or coat NPs, thereby altering their apparent bioavailability or excretion?

The compositions of the diets presented in Tables 4–6 do not appear to provide meaningful insight. Instead, the authors should offer more mechanistic evidence related to NP metabolism or elimination

Reviewer #2

(Remarks to the Author)
Reviewers' Comments
The paper analyzes an important dietary intervention using fiber to reduce the intestinal absorption of nanoplastics (NPs). The authors employ innovative confocal imaging methods to assess NP accumulation in the intestinal tract. The findings are relevant and innovative for the field of environmental toxicology.
Comments/Suggestions
• Manuscript structure – I suggest that the authors reorganize the manuscript. It would be clearer if it were divided into Background, Results, Discussion, Conclusion, and Methods sections. In its current form, the manuscript is difficult to follow.
• NP analysis in feces – The authors state that they analyzed NP levels in mouse feces; however, Figures 4C and 4D only show NP concentrations in different segments of the gastrointestinal tract, not in the feces. This dataset appears to be missing. Please include the fecal NP data in the same graphs (Figure 4C and 4D).
• Group inconsistency – The authors said in Methods that 72 mice were divided in 3 groups at 7 days treatment and 3 groups 28 days, both with SD, MFD and HFD. However, some analysis the authors used NC group. What NC group means? This group is not included in methods. The authors should clarify this better.
• Intestinal inflammation and barrier genes – In Figure 4, the authors report epithelial flattening and crypt loss in the intestines of SD mice, suggesting inflammation and intestinal barrier deterioration caused by NP exposure. I recommend assessing inflammatory genes (Tnfα, Il1b, Il6) and intestinal barrier-related genes (Cldn1, Cldn2, Cldn3, Ocln) across groups and time points. These results would complement the histological and ultrastructural findings.
• Liver histology – Since the authors collected liver samples for histology, it would be valuable to analyze liver H&E staining across groups. Dietary fiber in the intestine could potentially protect the liver from NP exposure. The authors may also consider performing F4/80 immunohistochemistry to assess immune cell infiltration and Sirius Red staining to evaluate fibrosis.
• Gut–liver axis – The observation that NP particles were detected only in the liver may be related to the gut–liver axis. The

authors should discuss this finding in the context of gut–liver axis physiology and NP translocation mechanisms. The authors also should indicate how many mice presented NP within the liver. If the authors used 12 mice in each group, how many of these mice NP was identified inside the liver.
• Statistical analysis – The authors didn`t mention which normality test was used to assume normal data. Moreover, the authors said they used One-Way ANOVA (parametric test); however, they do not include which post-test was used. It was Tukey test? Please include this.

Version 1:

Reviewer comments:

Reviewer #1

(Remarks to the Author)
1. The imaging strategy is technically sound and enables visualization of nanoplastic transit in vivo. However, the study remains largely descriptive. The conclusion that dietary fiber enhances nanoplastic clearance is based primarily on imaging observations, while the proposed mechanisms, including cellulose adsorption, accelerated intestinal transit, increased mucus secretion, and reduced epithelial contact, are only speculative and lack experimental validation.

2. The role of the gut microbiota is another major gap. Because many physiological effects of dietary fiber are mediated through microbial communities and their metabolites, microbiota-related analyses, such as 16S rRNA sequencing, metagenomics, SCFA, or bile acid profiling, should have been included. Without microbiota depletion or germ-free models, it remains unclear whether the observed effects are driven by dietary fiber directly or indirectly through changes in the gut microbiota.

3. The assessment of tissue accumulation relies entirely on NIR fluorescence without independent validation. Fluorescence signals alone cannot exclude potential artifacts caused by dye leakage, degradation, or redistribution. Confirmation using complementary techniques, such as Py-GC/MS, Raman spectroscopy, or FTIR, would provide more convincing evidence that the detected signals represent intact nanoplastics.

4. The translational relevance of the study is also limited. The high-fiber diet contained 50% dietary fiber, which is far beyond levels achievable in a normal human diet and may influence multiple physiological parameters. Validation using more realistic dietary fiber levels would strengthen the applicability of the findings to human nutrition and public health.

Reviewer #2

(Remarks to the Author)
This manuscript by Dr. Zeng and colleague's addresses an important question and observations regarding nanoparticle toxicity. This represents a submission with extensive changes made in response to the original review. The additions have significantly strengthened the frame work of the manuscript while addressing most of the critical concerns.

Open Access This Peer Review File is licensed under a Creative Commons Attribution 4.0 International License, which permits use, sharing, adaptation, distribution and reproduction in any medium or format, as long as you give appropriate credit to the original author(s) and the source, provide a link to the Creative Commons license, and indicate if changes were made.
In cases where reviewers are anonymous, credit should be given to 'Anonymous Referee' and the source.
The images or other third party material in this Peer Review File are included in the article's Creative Commons license, unless indicated otherwise in a credit line to the material. If material is not included in the article's Creative Commons license and your intended use is not permitted by statutory regulation or exceeds the permitted use, you will need to obtain permission directly from the copyright holder.
To view a copy of this license, visit https://creativecommons.org/licenses/by/4.0/

Response to Reviewers' Comments

Dear editor and reviewers: Thank you so much for the revision opportunity. New data are presented in Fig. 5g-5i, Fig. 6, Fig. 7, Fig. S1, Fig. S2, and Fig. S6 in the revised manuscript. Fig. 3b-3d were re-edited. All the revisions are in blue color in the context. Detailed point-by-point responses to all review comments are listed as follows.

Reviewer: 1

1. *General comments:*

I do not agree that the authors have demonstrated any important or novel findings in this study. In fact, no mechanistic insights were discussed or elucidated throughout the manuscript.

Response/action: We agree with the reviewer that the mechanism part should be strengthened. We have substantially revised the manuscript and added discussions on mechanisms based on both physicochemical experiments and histopathological data (Figures 6 and 7 in the revised manuscript) to clarify how cellulose modulates the gastrointestinal fate of nanoplastics (NPs).

(i) Physical mechanism

In response to the reviewer's concerns, we have added a paragraph to the revised manuscript to discuss the physical mechanism (lines 266–279):

"The pristine cellulose, which was added to produce high fiber diet, was used to elucidate the physical mechanism of dietary fiber in mitigating exposure risk of NPs *in vitro*. The results indicated that the digestion process significantly increased the NPs-harvesting property of cellulose (Fig. 6a). Water-treated cellulose could hardly remove NPs from water while digested cellulose could efficiently remove NPs from water and settle down (Fig. 6b). Interestingly, NPs did not form homogeneous complexes with cellulose, but formed some local high-concentration regions (Fig. 6c). This was further confirmed by SEM images, which show both encapsulation and local adhesion (Fig. 6d). There was no significant change in chemical functional groups (Fig. 6e). Notably, significant morphological change was observed in digested cellulose, which showed more abundant porous microstructures than pristine cellulose (Fig. 6f). The specific surface area of digested cellulose increased up to 10 times that of pristine cellulose (from 0.8898 to 8.9019 m^2 g^{-1}) (Fig. 6g). The dramatically increased surface area and enhanced porous structure might directly result in harvest of NPs by digested fiber, which decreases the bioavailability of NPs."

(ii) Histopathological mechanism

We have added another paragraph to examine the histopathological mechanism in the revised manuscript (lines 300–309):

"The histopathological mechanism was further studied on dietary fiber in mitigating the exposure risk of NPs. Long term exposure to low concentration NPs via food intake induced inflammation (high level IL-6) of small intestinal villus (SD group; Fig. 7a and Fig. S2), which was significantly alleviated by including dietary fiber in diets. Moreover, the decreased occludin expression indicates a weakened intra-cellular binding force (Fig. 7b), facilitating the transport of NPs via the gut-liver axis. In addition, a high-fiber diet generates longer and denser intestinal villus, which serves as a powerful shield to NPs. Dietary fiber possesses both physical encapsulation-adhesion and biological villous shield capabilities in mitigating exposure risk of NPs (Fig. S3)."

Major comments:

2. *Although the authors quantified NP accumulation in various organs, they did not analyze NP levels in blood or feces during the treatment period. Without these data, it is difficult to determine whether the dietary intervention truly promoted NP excretion.*

<u>Response/action</u>: Thank you very much for your valuable concern/suggestion. In the original manuscript, we reported the fecal excretion rates of NIR-NPs rather than the fecal NP concentrations because the feces amount varies a lot in SD and HFD groups. Adding dietary fiber significantly increased the feces. The quantitative fecal NP concentration data during the 0–24 h post-administration period are presented in a newly created Figure S1 in the revised manuscript. The results are added in the revised manuscript (lines 127–129):

"The total amount of NIR-NPs recovered from feces within 0–24 h was higher in the MFD (16 ± 0.45 μg) andHFD (19 ± 0.75 μg) groups than in the SD group (12 ± 0.66 μg) (Fig. S1)."

We acknowledge that blood NP levels would provide additional information regarding systemic exposure and transport. However, blood samples were not collected in the original experimental design. Therefore we are unable to provide blood NP concentration data. In addition, very low NP concentration was used in the present study. Considering the low transport speed, we feel that it would be a great challenge to dynamically detect NPs in blood.

3. *In Figure S4, fecal NP levels were measured only in the standard diet group. The authors should explain why no measurements were conducted for the other treatment groups.*

<u>Response/action</u>: Thank you very much for your insightful opinions and suggestions. Figure S4 (now Figure S6 in the revised manuscript) was intended to show the representative calibration/working curves used for quantifying NP concentrations in fecal and gastrointestinal samples, rather than to indicate that fecal NP levels were measured only in the standard diet group. In fact, NP concentrations were measured in all treatment groups, including the SD, MFD, and HFD group. Because each sample type and treatment group required its own matrix-matched calibration curve to minimize potential interference from fecal or gastrointestinal contents, we generated corresponding working curves for different groups and tissues.

To address this misunderstanding, we have revised Figure S4 (now Figure S6) and to include the calibration/working curves for all treatment groups. The revised supplementary figure more clearly demonstrates that NP quantification was performed using appropriate calibration curves for each group and sample matrix. We have also revised the figure legend and Methods section to clarify that fecal NP concentrations were quantified in all treatment groups using matrix-matched calibration curves in the revised manuscript (lines 404–407):

"NP concentrations in fecal and gastrointestinal samples from all dietary groups were quantified using matrix-matched fluorescence calibration curves. Representative standard curves for each dietary group and sample matrix are provided in Figure S6."

4. *Is it possible that the fiber diet may physically entrap or coat NPs, thereby altering their apparent bioavailability or excretion?*

Response/action: Your valuable suggestion is greatly appreciated. We have provided results from additional mechanistic experiments, which support the notion that cellulose can physically capture NPs through surface adhesion and pore-mediated entrapment. The result is added in the revised manuscript (lines 269–273):

"Water-treated cellulose could hardly remove NPs from water while digested cellulose could efficiently remove NPs from water and settle down (Fig. 6b). Interestingly, NPs did not form homogeneous complexes with cellulose, but formed some local high-concentration regions (Fig. 6c). This was further confirmed by SEM images, which show both encapsulation and local adhesion (Fig. 6d)."

5. *The compositions of the diets presented in Tables 4–6 do not appear to provide meaningful insight. Instead, the authors should offer more mechanistic evidence related to NP metabolism or elimination.*

Response/action: Thank you very much for your insightful opinions. Tables S4–S6 mainly provide the detailed formulations of the experimental diets. These tables were included to ensure transparency and reproducibility of the dietary intervention, particularly regarding different cellulose contents among the SD, MFD, and HFD groups. Both physicochemical experiments and histopathological data (Figures 6 and 7) have been added to clarify how cellulose modulates the gastrointestinal fate of nanoparticles. Dietary fiber possesses the capability of physical encapsulation-adhesion and serves as a biological villous shield in mitigating exposure risk of NPs. (lines 266–279 and lines 300–309 in the revised manuscript).

Reviewer: 2

General comments:

The paper analyzes an important dietary intervention using fiber to reduce the intestinal absorption of nanoplastics (NPs). The authors employ innovative confocal imaging methods to assess NP accumulation in the intestinal tract. The findings are relevant and innovative for the field of environmental toxicology.

Response/action: Thank you very much for your positive feedback on our study and findings. Detailed point-by-point responses to all review comments are listed as follows.

Major comments:

1. *Manuscript structure – I suggest that the authors reorganize the manuscript. It would be clearer if it were divided into Background, Results, Discussion, Conclusion, and Methods sections. In its current form, the manuscript is difficult to follow.*

Response/action: Thank you very much for your suggestion. We have revised the format according to the guidelines of *Nature Communications*.

2. *NP analysis in feces – The authors state that they analyzed NP levels in mouse feces; however, Figures 4C and 4D only show NP concentrations in different segments of the gastrointestinal tract, not in the feces. This dataset appears to be missing. Please include the fecal NP data in the same graphs (Figure 4C and 4D).*

Response/action: Thank you for your valuable comment. In the original manuscript, we reported the fecal excretion rate of NIR-NPs rather than the fecal NP concentration because the feces amount varies widely in the SD and HFD groups. Adding dietary fiber significantly increased the amount of feces. Fecal NP data were converted into fecal excretion rates and therefore were not directly shown as fecal NP concentrations in the corresponding figures. We agree that presenting fecal NP concentrations more clearly would help readers better evaluate NP elimination. In the revised manuscript, we have added the fecal NP concentration data to the relevant figure panels (Figure S2). These data show that dietary cellulose increased fecal NP recovery and promoted NP elimination, which is consistent with the reduced NP retention observed in different gastrointestinal segments. The discussion is added in the revised manuscript (lines 301–303):

"Long-term exposure to low concentration NPs via food intake induced inflammation (high level IL-6) of small intestinal villus (SD group; Fig. 7a and Fig. S2), which was significantly alleviated by including dietary fiber in diets"

3. *Group inconsistency – The authors said in Methods that 72 mice were divided in 3 groups at 7 days treatment and 3 groups 28 days, both with SD, MFD and HFD. However, some analysis the authors used NC group. What NC group means? This group is not included in methods. The authors should clarify this better.*

Response/action: Thank you very much for your questions. In the original text, "NC" referred to the control group. As this acronym was not clearly defined in the Methods section and could be confused with the dietary intervention groups, we have replaced "NC" with "Control" throughout the revised manuscript (line 203) and have clarified the group definitions in the Methods section. The dietary intervention groups remain SD, MFD, and HFD, corresponding to standard diet, medium-fiber diet, and high-fiber diet, respectively.

4. *Intestinal inflammation and barrier genes – In Figure 4, the authors report epithelial flattening and crypt loss in the intestines of SD mice, suggesting inflammation and intestinal barrier deterioration caused by NP exposure. I recommend assessing inflammatory genes (Tnfα, Il1b, Il6) and intestinal barrier-related genes (Cldn1, Cldn2, Cldn3, Ocln) across groups and time points. These results would complement the histological and ultrastructural findings.*

Response/action: Your valuable recommendations are greatly appreciated. We have added the inflammatory marker IL-6 and the tight junction protein occludin in intestinal tissues from different dietary groups and time points in Figure 7 and Figure S2. Discussions are added in the revised manuscript (lines 300–309):

"The histopathological mechanism was further studied on dietary fiber in mitigating the exposure risk of NPs. Long term exposure to low concentration NPs via food intake induced inflammation (high level IL-6) of small intestinal villus (SD group; Fig. 7a and Fig. S2), which was significantly alleviated by including dietary fiber in diets. Moreover, the decreased occludin expression indicates disruption of intercellular tight junctions and impairment of the intestinal epithelial barrier (Fig. 7b), facilitating the transport of NPs via the gut-liver axis. In addition, a high-fiber diet generates longer and denser intestinal villus, which serves as a powerful shield to NPs. Dietary fiber possesses both physical encapsulation-adhesion and biological villous shield capabilities in mitigating exposure risk of NPs (Fig. S3)."

5. *Liver histology – Since the authors collected liver samples for histology, it would be valuable to analyze liver H&E staining across groups. Dietary fiber in the intestine could potentially protect the liver from NP exposure. The authors may also consider performing F4/80 immunohistochemistry to assess immune cell infiltration and Sirius Red staining to evaluate fibrosis.*

Response/action: Thank you very much for your valuable comment. In response to the review comment, we performed H&E staining, F4/80 immunostaining, and Sirius Red staining on the paraffin-sealed liver tissues to evaluate whether dietary fiber intervention could alleviate NP-associated liver injury. Discussions are added in the revised manuscript (lines 242–248):

"Hematoxylin and eosin (H&E) staining of liver tissues indicates that the SD group showed evident hepatic inflammatory changes, including focal inflammatory cell infiltration and disrupted local hepatic architecture after 28 days of NP exposure (Fig. 5g). Consistently, F4/80 staining was increased in the SD group, indicating enhanced macrophage infiltration and hepatic immune activation (Fig. 5h). Sirius Red staining also showed limited collagen-positive signals in the SD group, suggesting mild extracellular matrix deposition, although no severe fibrosis was observed (Fig. 5i)."

6. *Gut–liver axis – The observation that NP particles were detected only in the liver may be related to the gut–liver axis. The authors should discuss this finding in the context of gut–liver axis physiology and NP translocation mechanisms. The authors also should indicate how many mice presented NP within the liver. If the authors used 12 mice in each group, how many of these mice NP was identified inside the liver.*

Response/action: Your insightful opinions are well taken. Six mice were used in each control and NP exposure group, including three for in vivo imaging analysis and three for histological and fluorescence section analysis. For tissue section analysis, four liver sections were examined from each mouse. NP fluorescence signals were observed only in the liver sections of the SD group. Specifically, positive NP signals were detected in 3/3 SD mice used for tissue section analysis, whereas no obvious NP fluorescence signal was observed in the liver sections from the MFD or HFD groups. The results and discussions are added in the revised manuscript (lines 240–241):

"NIR-NPs were found within liver tissue sections (3/3) via fluorescence microscopic imaging (Figs. 5d–5f)."

After oral exposure, NPs that translocate across the intestinal epithelium may enter the portal venous system and preferentially accumulate in the liver, where they may be retained by liver sinusoidal endothelial cells and hepatic macrophages/Kupffer cells. The relevance of the gut-liver axis in regulating the hepatic delivery of gut-derived dietary components has been widely recognized. Recent studies also suggested that micro-/nanoplastics can affect liver homeostasis through intestinal barrier disruption, inflammation, microbiota changes, and gut-liver axis-mediated translocation. Based on the new mechanism data in Figures 6 and 7, dietary fiber possesses both physical encapsulation-adhesion and biological villous shield capability in mitigating exposure risk of NPs. It hinders the gut–liver axis transport of NPs from the beginning of absorption. Discussions are added in the revised manuscript (lines 266–279 and lines 300–309):

"The pristine cellulose, which was added to produce high fiber diet, was used to elucidate the physical mechanism of dietary fiber in mitigating exposure risk of NPs *in vitro*. The results indicated that the digestion process significantly increased the NPs-harvesting property of cellulose (Fig. 6a). Water-treated cellulose could hardly remove NPs from water while digested cellulose could efficiently remove NPs from water and settle down (Fig. 6b).

Interestingly, NPs did not form homogeneous complexes with cellulose, but formed some local high-concentration regions (Fig. 6c). This was further confirmed by SEM images, which show both encapsulation and local adhesion (Fig. 6d). There was no significant change in chemical functional groups (Fig. 6e). Notably, significant morphological change was observed in digested cellulose, which showed more abundant porous microstructures than pristine cellulose (Fig. 6f). The specific surface area of digested cellulose increased up to 10 times that of pristine cellulose (from 0.8898 to 8.9019 m^2 g^{-1}) (Fig. 6g). The dramatically increased surface area and enhanced porous structure might directly result in harvest of NPs by digested fiber, which decreases the bioavailability of NPs." (lines 266–279)

"The histopathological mechanism was further studied on dietary fiber in mitigating the exposure risk of NPs. Long-term exposure to low concentration NPs via food intake induced inflammation (high level IL-6) of small intestinal villus (SD group; Fig. 7a and Fig. S2), which was significantly alleviated by including dietary fiber in diets. Moreover, the decreased occludin expression indicates disruption of intercellular tight junctions and impairment of the intestinal epithelial barrier (Fig. 7b), facilitating the transport of NPs via the gut-liver axis. In addition, a high-fiber diet generates longer and denser intestinal villus, which serves as a powerful shield to NPs. Dietary fiber possesses both physical encapsulation-adhesion and biological villous shield capabilities in mitigating exposure risk of NPs (Fig. S3). It hinders the gut-liver axis transport of NPs from the beginning of absorption." (lines 300–309)

7. *Statistical analysis – The authors didn`t mention which normality test was used to assume normal data. Moreover, the authors said they used One-Way ANOVA (parametric test); however, they do not include which post-test was used. It was Tukey test? Please include this.*

Response/action: Thank you very much for you helpful reminder and concerns. In the present study, data were analyzed using one-way ANOVA followed by Tukey's multiple comparisons test. The result is added in lines 470–471 in the revised manuscript, "One-way analysis of variance followed by Tukey's multiple comparisons test was employed for data comparison".

Reviewer: 1

1. *The imaging strategy is technically sound and enables visualization of nanoplastic transit in vivo. However, the study remains largely descriptive. The conclusion that dietary fiber enhances nanoplastic clearance is based primarily on imaging observations, while the proposed mechanisms, including cellulose adsorption, accelerated intestinal transit, increased mucus secretion, and reduced epithelial contact, are only speculative and lack experimental validation.*

Response/action: Thank you very much for your valuable comments. First of all, we feel that our study is a bit more than being descriptive. The in vitro simulated digestion experiments well elucidate the interaction between cellulose and nanoplastics, and the results support the proposed encapsulation/adhesion mechanism that decreases nanoplastic bioavailability (lines 188–203, 266–281 and Fig. 7 in the revised manuscript). Such in vitro experiments have been frequently utilized in previous mechanism studies.

Second, we have also incorporated tissue-level evidence to support the protective effect of dietary fiber on the intestinal barrier. Histopathological and immunofluorescence analyses show that dietary fiber alleviates nanoplastics-induced intestinal inflammation, preserves villus structure, maintains occludin expression, and enforces barrier protection (lines 239–265 and Fig. 5 in the revised manuscript). These findings suggest that dietary fiber may reduce epithelial contact with nanoplastics and limit their transport across the intestinal barrier and along the gut-liver axis. Together with the quantitative fecal recovery and gastrointestinal retention data, these in vitro and histological results strengthen the conclusion that dietary fiber promotes nanoplastic clearance and reduces intestinal exposure, which does not rely on descriptive imaging observations only.

2. *The role of the gut microbiota is another major gap. Because many physiological effects of dietary fiber are mediated through microbial communities and their metabolites, microbiota-related analyses, such as 16S rRNA sequencing, metagenomics, SCFA, or bile acid profiling, should have been included. Without microbiota depletion or germ-free models, it remains unclear whether the observed effects are driven by dietary fiber directly or indirectly through changes in the gut microbiota.*

Response/action: Thank you very much for insightful opinions. We agree that the gut microbiota and its metabolites may contribute to the effects of dietary fiber. We have therefore revised the Discussion to present microbiota-dependent pathways as a plausible complementary explanation based on previous evidence. The following paragraph has been added to the revised manuscript (lines 282–298):

"The gut microbiota and its metabolites may represent an additional pathway through which dietary fiber modulates the intestinal fate of NPs. Dietary fiber is an important substrate

shaping the composition and metabolic activity of the gut microbiota, and microbial fermentation of dietary fiber generates short-chain fatty acids, including acetate, propionate, and butyrate, which contribute to the maintenance of the mucus layer, epithelial barrier function, and mucosal immune homeostasis. Conversely, prolonged dietary fiber deprivation can shift microbial substrate utilization toward host-derived mucin glycans, resulting in erosion of the mucus barrier and increased epithelial accessibility. These effects may be particularly relevant to NP exposure because the integrity of the mucus and epithelial barriers determines the accessibility of luminal particles to the intestinal epithelium and their subsequent uptake. Polystyrene NPs have also been reported to perturb microbiota–host interactions, and interfere with the host-bacteria signal communication mediated by extracellular vesicles, accompanied by reduced expression of barrier-associated proteins and increased intestinal permeability. Thus, dietary fiber-induced modulation of the gut microbiota and its metabolic environment may further reduce sustained mucosal exposure to NPs by supporting mucus–epithelial barrier homeostasis and gastrointestinal physiological function."

We have also explicitly acknowledge this viewpoint in the Limitations (lines 333–335):

"In addition, modulation of the gut microbiota by dietary fiber may constitue a potential pathway affecting NP clearance and reduced intestinal exposure, requiring further investigation"

3. *The assessment of tissue accumulation relies entirely on NIR fluorescence without independent validation. Fluorescence signals alone cannot exclude potential artifacts caused by dye leakage, degradation, or redistribution. Confirmation using complementary techniques, such as Py-GC/MS, Raman spectroscopy, or FTIR, would provide more convincing evidence that the detected signals represent intact nanoplastics.*

Response/action: Thank you very much for your important opinions. The near-infrared nanoplastics used in the present study were prepared by an emulsion-solvent swelling method, in which the fluorescent dyes were encapsulated within polymer particles rather than being adsorbed onto the particle surface. Free dyes and residual impurities were removed by dialysis. This internal encapsulation strategy reduces the risk of dye desorption or leakage compared with surface-labelled commercial particles (lines 91–97 in the revised manuscript). In addition, dye leakage would be expected to produce diffuse background fluorescence or broadly distributed tissue signals. The tissue sections in the present study showed discrete punctate, particle-like fluorescence signals, which are consistent with particle-associated fluorescence, but not with free dye leakage, degradation, or homogeneous redistribution.

Concentrations of tissue NPs were determined using NIR fluorescence based on high-quality standard curves (Fig, S6). The results were further verified using fluorescent microscope directly showing the exact position of NPs in tissues. Py-GC/MS is an accurate

quantitative method but is unable to provide any information about the locality of NPs. This is the unique advantage of our study.

However, fluorescence-based measurements alone cannot independently confirm the presence of intact NPs. The following sentence has been added to the revised manuscript (lines 326–330), "The present study assessed tissue-associated NPs primarily using NIR fluorescence. Although this approach enabled sensitive spatiotemporal tracking, complementary analytical methods, such as pyrolysis-GC/MS, Raman spectroscopy, Fourier-transform infrared spectroscopy, or stable-isotope-based quantification, would provide independent confirmation of the chemical identity and tissue burden of NPs".

4. *The translational relevance of the study is also limited. The high-fiber diet contained 50% dietary fiber, which is far beyond levels achievable in a normal human diet and may influence multiple physiological parameters. Validation using more realistic dietary fiber levels would strengthen the applicability of the findings to human nutrition and public health.*

Response/action: Thank you very much for your significant feedback. We agree that the translational relevance of the dietary intervention should be interpreted cautiously. In the present study, the standard-fiber diet (5%) and medium-fiber diet (15%) were used to represent low and moderate fiber intake conditions, whereas the high-fiber diet was included as reference to provide proof-of-concept evidence (lines 394–396 in the revised manuscript). Our findings indicate that increasing dietary fiber intake can reduce nanoplastic retention and intestinal exposure in mice, thereby helping to preserve intestinal health. Although these results should not be directly translated into dietary recommendations for humans, they provide a useful experimental basis for considering dietary fiber as a potential strategy to mitigate nanoplastic exposure. The following sentence has been added to the revised manuscript (lines 335–343), "Finally, the present study was conducted exclusively with male mice, and the dietary-fiber levels used, particularly the 50% fiber diet, substantially exceed those typical of human diets. The high-fiber condition should therefore be regarded as a proof-of-concept intervention rather than a directly translatable dietary regimen. Although the 15% fiber diet represents a more moderate intervention, it likewise cannot be directly translated into a human dietary recommendation. Further studies are needed to incorporate both sexes, physiologically relevant fiber levels, and ultimately human populations before these findings can be translated into dietary recommendations or public-health strategies."

Reviewer: 2

This manuscript by Dr. Zeng and colleague's addresses an important question and observations regarding nanoparticle toxicity. This represents a submission with extensive changes made in response to the original review. The additions have significantly strengthened the frame work of the manuscript while addressing most of the critical concerns.

Response/action: We appreciate the reviewer's constructive comments, which have helped us to strengthen the manuscript.
